# Genetic parameters and quantitative trait loci analysis associated with body size and timing at metamorphosis into glass eels in captive-bred Japanese eels (*Anguilla japonica*)

**DOI:** 10.1371/journal.pone.0201784

**Published:** 2018-08-29

**Authors:** Kazuharu Nomura, Atushi Fujiwara, Yuki Iwasaki, Issei Nishiki, Aiko Matsuura, Akiyuki Ozaki, Ryusuke Sudo, Hideki Tanaka

**Affiliations:** 1 National Research Institute of Aquaculture, Japan Fisheries Research and Education Agency, Minami-ise, Mie, Japan; 2 National Research Institute of Fisheries Science, Japan Fisheries Research and Education Agency, Yokohama, Kanagawa, Japan; Fred Hutchinson Cancer Research Center, UNITED STATES

## Abstract

The Japanese eel (*Anguilla japonica*) is among the most important aquaculture fish species in Eastern Asia. The present study aimed to identify the genetic parameters underlying body size and the timing at metamorphosis from leptocephali to glass eels in captive-bred Japanese eels, with the intent to foster sustainable development. Larvae from a partly factorial cross (14 sires × 11 dams) were reared until the point of metamorphosis into glass eels. In these organisms, we observed moderate heritability and mild genetic correlations among traits related to body size (h^2^ = 0.16–0.33) and timing at metamorphosis (h^2^ = 0.36–0.41). In an F_1_ full-sib family, quantitative trait loci (QTL) mapping for these traits identified one significant (genome-wide *P* < 0.05) and five suggestive QTLs (chromosome-wide *P* < 0.05). These results suggest that in the Japanese eel, metamorphic traits exhibit a polygenic genetic structure comprising many QTLs with small effects. In addition, we updated the genetic linkage map for the Japanese eel and integrated it with our newly constructed *de novo* genome assembly. The information and tools generated from this study will contribute to the development of freshwater eel genetics and genomics.

## Introduction

The Japanese eel (*Anguilla japonica*) is considered one of the most important aquaculture fish species in East Asian countries. However, the eel culture industry relies completely on the capture of wild glass eels in estuaries for stocking purposes. Accordingly, eel culture has recently experienced serious restrictions caused by global shortages and rising prices of wild glass eels[1]. Therefore, the establishment of artificial glass eel production techniques, such as generating seedlings for aquaculture to reduce the demand for wild fish, is imperative to sustainable development in the eel culture industry.

Several successful studies of the life cycle of Japanese eels in captivity have been reported during the past 50 years[2,3]. Hormonal treatments have been used successfully to obtain artificially matured gametes[4], and the development of appropriate rearing techniques have led to the production of viable leptocephali[5] and glass eels[3]. In the year 2010, the entire life cycle of *A*. *japonica* was finally achieved in captivity (i.e., without wild inputs) with the generation of second-generation (F_2_) organisms[6]. Although further improvements are needed to ensure that glass eels can be produced commercially in sufficient numbers to meet the demands of the eel culture industry[7], recent advances have made it possible to artificially produce progeny for genetic studies[8–11] and to improve production performance using selective breeding programs.

The artificial mass-production of glass eels is hindered by a long larval period before metamorphosis, as well as the low survival rate of larvae in captivity[12]. Previous otolith microstructure analyses suggested that wild *A*. *japonica* leptocephali metamorphose at 110–170 days post-hatching (dph) under natural conditions[13–17], whereas captive-bred leptocephali metamorphose at 160–450 dph under the rearing conditions used in the present study. This large gap may be attributable to nutritional differences between the artificial diet and the food available in the natural environment. However, considerable phenotypic variation between individuals reared in a very similar environment may indicate genetic variation. Furthermore, individual differences during the larval period are likely attributable to both the larval growth rate and body size at the start of metamorphosis.

The estimation of genetic parameters (e.g., heritability and genetic correlations) is an important factor in decisions regarding the design and implementation of selective breeding programs. Accordingly, to assess the utility of selective breeding for metamorphic traits in Japanese eel, the heritabilities of those traits have to be known. Studies of the genetic architecture related to vertebrate larval metamorphosis timing and/or body size have been conducted in amphibians[18–20]. By contrast, few studies have addressed these factors in fishes. In this study, we quantified the genetic parameters associated with timing and several morphological traits associated with metamorphosis from leptocephali to glass eels. Furthermore, we report the results of a genome-wide quantitative trait locus (QTL) scan associated with metamorphosis in captivity. Our results represent the first step towards identifying the genetic architecture of traits important to the mass production of glass eels in the Japanese eel. Furthermore, in the present study, we updated the double digest restriction-site associated DNA sequencing technology (ddRAD)-based genetic linkage map and draft genome of the Japanese eel, and integrated this information to identify the chromosomal order of the scaffolds. These tools are expected to contribute to the development of *A*. *japonica* genetics and genomics.

## Materials and methods

### Ethics statement

This project was conducted in accordance with the Guidelines for Animal Experimentation of the National Research Institute of Aquaculture (NRIA) in Mie, Japan. All animal procedures were approved by the Institutional Animal Care and Use Committee of the NRIA.

### Factorial crosses and parental assignments for the estimation of genetic parameters

To estimate the genetic parameters associated with body size and timing at metamorphosis from leptocephali to glass eels in captive-bred *A*. *japonica*, we designed a partly factorial cross (14 sires x 11 dams) using artificial fertilization techniques in wild glass eels purchased from a commercial farm as follows. Half of the eels were feminized after consuming a diet containing estradiol-17β for 5 months, while the remaining eels were fed a normal diet. All eels were cultivated in freshwater for 2–3 years, and subsequently acclimated to seawater at the NRIA. Fourteen male eels were repeatedly injected with recombinant eel luteinizing hormone (rLH, ARK Resource, Kumamoto, Japan) to induce maturation[21], after which semen was collected from each male and cryopreserved in liquid nitrogen until insemination[22]. Eleven female eels were first injected with salmon pituitary extract (SPE), followed by 17α-hydroxyprogesterone (Sigma, St. Louis, MO, USA). Subsequently, eggs were collected from ovulating females by gentle stripping. The eggs from each dam (100–300 g) were divided equally into 10–11 batches that were inseminated individually with thawed cryopreserved sperm from each sire. After 5 min, fertilized eggs in each batch were pooled (i.e., maternal half-sib) and incubated in a 180-l polycarbonate tank supplied with filtered seawater (1 L/min) at 25.0 ± 0.5°C until 6 dph.

At 6 dph, approximately 1,000 larvae were stocked in each of one or two 20-l acrylic tanks supplied with filtered seawater (1 L/min) at 25.0 ± 0.5°C. Larval rearing regimens were applied according to previously described procedures[5]. Briefly, the larvae were fed an artificial diet composed of spiny dogfish (*Squalus acanthias* L., 1758) five times per day (0900, 1100, 1300, 1500, and 1700 hours). The hygienic rearing tanks were replaced every evening until 410 dph. This procedure was repeated 11 times during 2014–2016 to generate 11 maternal half-sib families in nine pairs of rearing tanks.

A total of 810 larvae were isolated from rearing tanks upon initiating metamorphosis to glass eels (pre-anal length/total length ratio decrease to ≤70%), anesthetized with 2-phenoxyethanol (400 ppm), and digitally photographed. Each larva was numbered, and the following phenotypic data were obtained: age at the start of metamorphosis (Age1), total length (TL1), pre-anal length (PAL1), and body depth (BD1). Larvae were reared individually in 250-ml plastic bottles supplied with filtered seawater (50 ml/min) at 25.0 ± 0.5°C; no food was provided until the morphological changes were completed. Approximately 2 weeks after the start of metamorphosis, 779 larvae that had completely transformed to glass eels were anesthetized, digitally photographed, and weighed. The following phenotypic values were recorded for each glass eel: age at the end of metamorphosis (Age2), total length (TL2), pre-anal length (PAL2), body depth (BD2), body weight (BW2), and growth rate (GR; TL2/Age2). Tail tissue (10–20 mg) was collected from each glass eel and preserved in 99.5% ethanol for DNA extraction.

DNA samples were extracted from 810 tail tissue samples of offspring and 25 fin samples of parental eels (14 sires, 11 dams). All individuals were genotyped according to our previously reported method[9,11]. One previously reported (Ajp-340) and nine newly developed short tandem repeat (STR) markers were analyzed (Ajp-716, Ajp-764, Ajp-770, Ajp-774, Ajp-775, Ajp-797, Ajp-810, Ajp-968, Ajp-981) (S1 Table). Parentage assignments were performed via the exclusion method, using PARFEX software[23].

### Estimation of genetic parameters

Uni- and multi-trait linear mixed models were applied respectively to estimate the heritabilities and correlations of all studied traits, using ASReml software v4.1 (VSN international Ltd, UK)[24]. In a matrix notation, the model is written as follows:

$$\text{y}=\text{Xb}+Z_{1}s+Z_{2}d+Z_{3}f+e,$$

where *y* is an observation vector for the studied traits; *X*, *Z*_*1*_, *Z*_*2*_, and *Z*_*3*_ are incidence matrices related respectively to the fixed, random sire, random dam, and random interaction sire by dam effects; *b* is the fixed effects vector (year/month of rearing start); and *s*, *d*, *f*, and *e* are the random sire, dam, interaction sire by dam, and residual effects vectors, respectively.

Using this model, trait heritabilities were estimated as follows:

$$h^{2}=4\sigma_{s}^{2}/\sigma_{p}^{2},$$

where $\sigma_{s}^{2}$ is the additive genetic sire variance, $\sigma_{d}^{2}$ is the additive genetic dam variance, $\sigma_{f}^{2}$ is the non-additive genetic variance, $\sigma_{e}^{2}$ is the residual variance, and $\sigma_{p}^{2}=\sigma_{s}^{2}+\sigma_{d}^{2}+\sigma_{f}^{2}+\sigma_{e}^{2}$.

Genetic and phenotypic correlations were estimated using multivariate linear mixed model with the above-described fixed and random effects. Correlations were calculated as the covariance divided by the product of the standard deviations of traits:

$$\text{r}=\frac{\sigma_{12}}{\sqrt{\sigma_{1}^{2}}\sqrt{\sigma_{2}^{2}}},$$

Where *σ*_12_ is the estimated additive genetic or phenotypic covariance between two traits, and $\sigma_{1}^{2}$ and $\sigma_{2}^{2}$ are the additive genetic or phenotypic variances of traits 1 and 2, respectively.

### Generation of a full-sib family for QTL mapping

The genetic architecture of the studied traits (i.e., the number, genome location and effects of QTLs) was studied using a mapping family involving a two-generation outbred pedigree comprising 287 F_1_ progeny from a single cross, as described by Kai et al. (2014)[9]. Maturation and fertilization were artificially induced and larval rearing was subsequently conducted as described above, with slight modifications. Twenty-one 5-l acrylic bowl-type tanks were each stocked with approximately 500 larvae (6 dph) (total: 10,500 larvae) and reared until metamorphosis began. After recording the Age1, 287 larvae that had started metamorphosis were isolated from a rearing tank and reared separately without food until the morphological changes were completed. Within approximately 2 weeks, 275 larvae had completely transformed to glass eels and were subsequently anesthetized, digitally photographed, and weighed. Each glass eel was numbered, and the following phenotypic values were recorded: Age2, TL2, PAL2, BD2, BW2, and GR (TL2/Age2). Tail tissue (10–20 mg) was collected from each glass eel and preserved in 99.5% ethanol for DNA extraction. A total of 240 individuals, including the two parents, were then used for linkage and QTL mapping analyses.

### ddRAD-seq analysis

Genomic DNA samples were extracted from 238 tail tissue samples of glass eels and two parental muscle samples using the DNeasy Blood & Tissue Kit (Qiagen, Venlo, Netherlands). The ddRAD libraries were essentially prepared as described in our previous paper [9], with slight modifications. Briefly, genomic DNA was digested using two restriction enzymes (*Sbf*I and *Msp*I) and ligated using P5 and P7 adapters with unique barcodes for individual identification. The ligates from approximately 30–50 individuals were pooled in equimolar proportions and size selected within a range of 350–450 bp (insert DNA from 200 to 300 bp) using BluePippin (Sage Science, Beverly, MA, USA). After size selection, the ddRAD libraries were amplified by 12-cycle PCR, purified using Agencourt AMPure XP beads (Beckman Coulter Inc., Brea, CA, USA), and sequenced on a NextSeq 500 platform (Illumina, Inc., San Diego, CA, USA) with single-end reads of 151 bp, according to the manufacturer's protocol.

Raw reads were filtered using the process_radtags program in Stacks v1.4[25] with the following options: restriction enzyme, *Sbf*I; rescue barcodes and RAD-tags; discard reads with low quality scores; remove any read with an uncalled base; and truncate final read length, 100 bp. The processed reads were mapped to the draft genome sequence[26] using BWA-MEM[27] with default parameters. The mapped reads were then filtered manually using the following criteria: MQ of >30; discard all reads with multiple hits; and minimum total alignment length percentage of >95%. Subsequently, the filtered reads were realigned using RealignerTargetCreator and IndelRealigner in the Genome Analysis Toolkit (GATK)[27]. Next, Unified Genotyper in the GATK was used to perform variant calling, and the raw single nucleotide polymorphisms (SNPs) were filtered using the following criteria: QD of <2.0, MQ of <40.0, MQRankSum of <−12.5, and ReadPosRankSum of <−8.0.

For each individual, the genotyping data were further filtered using the following criteria: DP of >30, GQ of >30, REF or ALT allele frequency of >0.25 at heterozygous sites, REF or ALT allele frequency of >0.95 at homozygous sites, sum of REF and ALT allele frequencies of >0.95, and a lack of Mendelian contradictions between the genotypes of parents and each progeny. For cases of multiple filtered SNPs in a ddRAD-tag, each SNP was manually phased using SAM file read mapping information. The resulting phased SNP genotyping data were then filtered as mentioned above and used for linkage mapping using the standard codes for a cross pollination (CP)-type progeny in JOINMAP 4.1 (Kyasma), which was designed to evaluate genotyping data from an outbreeding full-sib family[28]. The following five segregation types were genotyped: type “nn × np,” homozygous in the female and heterozygous in the male parent; “lm × ll,” heterozygous in the female and homozygous in the male parent; “hk × hk,” heterozygous in both parents with two shared alleles; “ef × eg,” heterozygous in both parents with two sex-specific alleles and one shared allele; and “ab × cd,” heterozygous in both parents with four sex-specific alleles.

### Linkage map construction

A genetic linkage map was constructed using the multipoint maximum likelihood mapping algorithm for the population type of CP in JONMAP 4.1. This mapping algorithm allows the simultaneous estimation of two separate maps for each parent, followed by integration into a single consensus map. Markers that were genotyped in at least 215 of 238 samples (call rate >90%), were grouped using the independence logarithm of the odds (LOD) option in JOINMAP, with a range of 6–10 LOD (minimum–maximum thresholds, respectively). Nineteen linkage groups (LGs) were defined by grouping tree branches with stable marker numbers over increasing consecutive LOD values. To confirm and synchronize each LG number with our previously published linkage map[9], we created a dataset that integrated previously and newly generated genotyping data for the 92 individuals common to both datasets and performed a separate linkage analysis. The corrected linkage map (*L*) length was estimated by multiplying the length of each linkage group by (*m*+1)/(*m*−1), where *m* is the number of markers in the linkage group[29]. The linkage map genome coverage (*c*) was then estimated as *c* = 1− *e*^*−2dn/L*^, where *d* is the average interval of markers, *n* is the number of markers, and *L* is as estimated above.

### QTL analysis

The traits were subjected to a simple-interval QTL mapping analysis using MapQTL 6 software (Kyasma). Among the 238 offspring subjected to linkage analysis, 48 were excluded from the QTL analysis because of unknown correspondences between genotyping and phenotypic data. The LOD score significance thresholds for each trait were determined using 1,000 permutation tests. QTLs with LOD scores exceeding the chromosome-wide and genome-wide LOD thresholds with a P < 0.05 were considered suggestive and significant, respectively.

### Draft genome sequencing and genetic map integration

**Fig 1:**
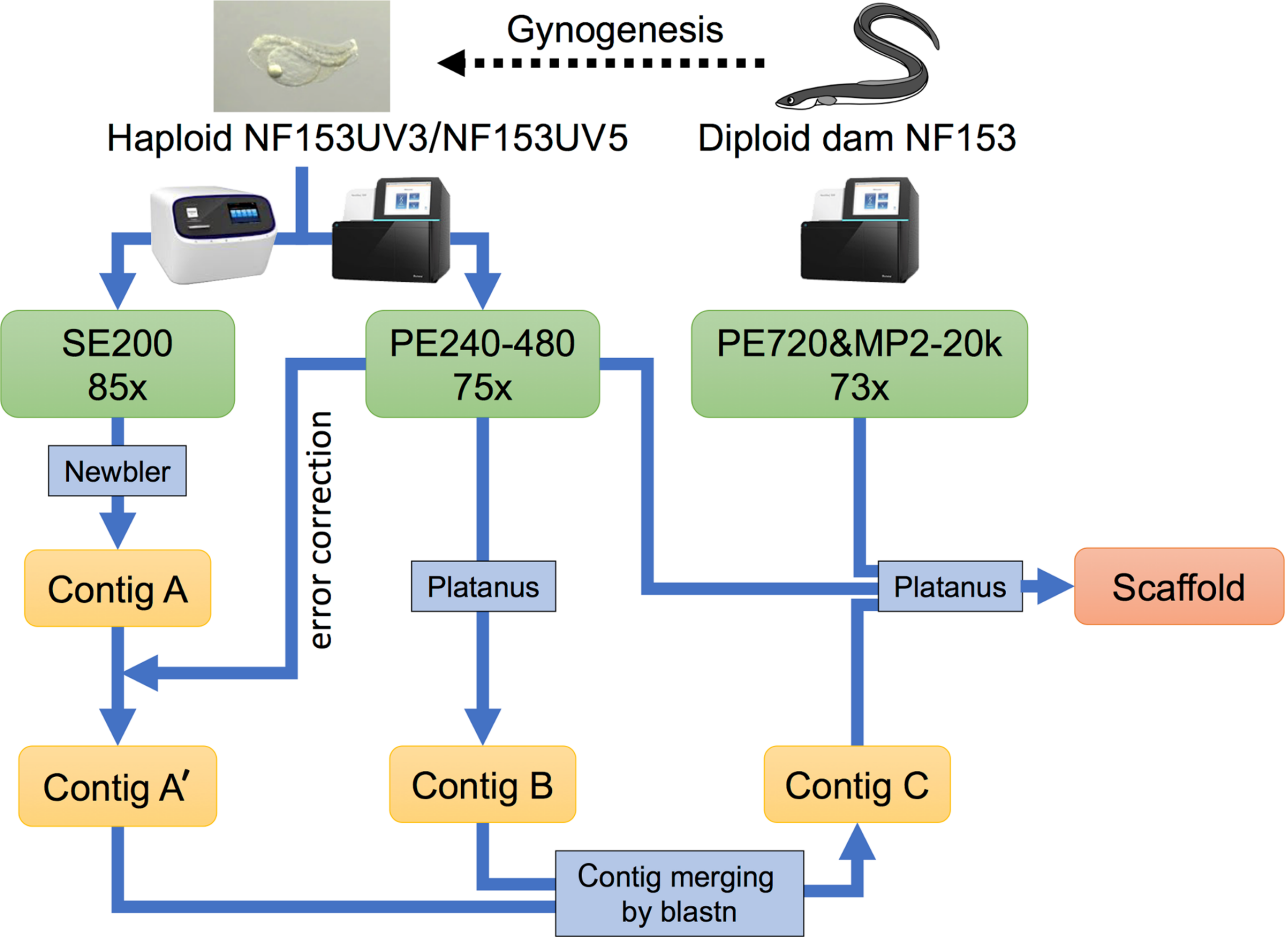
Flowchart describing the *de novo* genome assembly pipeline used in the present study.

Two gynogenetic haploid larvae generated by artificial insemination with UV-irradiated sperm, as described by Iwasaki et al (2016)[30], and their diploid dam were subjected to shotgun sequencing (Fig 1). Genomic libraries of 200-bp DNA fragments were prepared from haploid NF153UV5 using the Ion Xpress Plus gDNA Fragment Library kit (Life Technologies, Carlsbad, CA, USA) and sequenced on an Ion Proton system using the Ion P1 Sequencing 200 Kit v3 (Life Technologies) according to the manufacturer’s protocol. Genomic libraries of 240-, 360-, 480-bp DNA fragments from haploid NF153UV3 and 720-bp fragments from diploid dam NF153 were prepared using the TruSeq Nano DNA LT Sample Prep Kit (Illumina, Inc.). Mate pair libraries of 2-, 5-, 10-, and 20-kb DNA fragments were prepared from diploid dam NF153 using the Nextera Mate Pair Sample Prep Kit (Illumina, Inc.). These libraries were sequenced on a NextSeq 500 platform (Illumina, Inc.) with paired-end reads of 151 bp, according to the manufacturer's protocol. The obtained reads were pre-processed using CLC Genomics Workbench (CLC Bio, Aarhus, Denmark) as follows: i) duplicate reads were discarded using the Remove Duplicate program; ii) low-quality nucleotides and shorter reads were discarded (<150 bp in Ion Proton reads, <50 bp in Illumina reads) using the Trim Sequences program; and iii) 240-bp paired-end reads were merged using the Merge overlapping pairs program with the default parameters.

A *de novo* assembly was performed from pre-processed reads (S2 Table) as follows (Fig 1): i) reads from haploid NF153UV5 were assembled using Newbler ver 2.9 (Contig A); ii) reads from haploid NF153UV3 were mapped to Contig A using BWA-MEM, and the mapped consensus sequences were called (Contig A'); iii) reads from haploid NF153UV3 were assembled using Platanus[27] (Contig B); iv) specific sequences of Contig B against Contig A', identified using the Blast program[31,32], were added to Contig A’ to generate Contig C); and v) Contig C was scaffolded and gap-closed with all paired-end and mate pair reads using Platanus with the default parameters.

To identify the chromosomal order of scaffolds, we used ALLMAPS software[33] to anchor scaffolds split by the chimeric boundaries of genetic maker positions to three types of linkage maps: a newly developed consensus map and previously published male- and female-specific maps by Kai et al. (2014)[9]. Scaffolds anchored to multiple linkage groups were considered chimeric scaffolds that had been incorrectly generated during the genome assembly process, and were divided into two at the anchored marker positions at opposite ends of different linkage groups. The sequences of these boundary areas were considered ‘unplaced’.

The completeness of the genome assembly was estimated using CEGMA [34] and BUSCO (version 3.0.2) [35]. For CEGMA analysis, we used the 248 core vertebrate genes (CVGs). For BUSCO analysis, we used the vertebrate-specific orthologue catalogue (vertebrata_odb9) instead of actinopterygii_odb9, because Japanese eel is a primitive teleost.

## Results

### Phenotypic variation

The factorial and full-sib experimental crosses had similar phenotypic values for each trait (Tables 1 and S3 and S4). Age1 varied considerably within each experimental cross, with means ± standard deviations of 266.1 ± 51.5 dph in the factorial cross and 292.0 ± 52.0 dph in the full-sib family. The mortality rates from the start of rearing (6 dph) to metamorphosis were high in both experimental crosses. The average percentages of larvae that started metamorphosis were 4.8% (1.8%–12.5%/dam; data not shown) in the factorial cross and 2.7% in the full-sib family. Of the larvae that started metamorphosis, 31 (3.8%) and 10 (3.5%) larvae in the factorial cross and full-sib family, respectively, died before completing metamorphosis. Death was mainly attributable to symptoms of apparent oxygen deficiency, accompanied by a failure of metamorphosis progression.

**Table 1:** Number of data records (n), mean values, standard deviations (SDs), minimum and maximum values and range (Max–Min), and median values of metamorphic traits in two experimental crosses.

Traits (unit)	Experimental cross	*n*	Mean	SD	Min	Max	Range	Median
Age1	Factorial	810	266.1	51.5	163	407	244	257
(dph)	Full-sib	287	292.0	52.0	186	447	261	285
TL1	Factorial	810	54.19	5.00	34.48	69.86	35.38	54.22
(mm)	full-sib	ND	ND	ND	ND	ND	ND	ND
PAL1	Factorial	810	31.07	4.73	15.52	44.93	29.41	31.55
(mm)	full-sib	ND	ND	ND	ND	ND	ND	ND
BD1	Factorial	810	9.47	1.74	3.17	15.01	11.84	9.60
(mm)	full-sib	ND	ND	ND	ND	ND	ND	ND
Age2	Factorial	779	276.4	52.2	173	423	250	268
(dph)	full-sib	277	302.5	51.7	198	457	259	293
TL2	Factorial	779	53.17	4.83	30.16	68.86	38.70	53.08
(mm)	full-sib	277	53.11	4.77	37.20	63.59	26.39	53.91
PAL2	Factorial	779	20.44	2.36	13.67	44.20	30.53	20.14
(mm)	full-sib	277	20.89	2.04	15.43	35.33	19.90	20.90
BD2	Factorial	779	3.35	0.56	1.90	5.51	3.61	3.24
(mm)	full-sib	277	3.27	0.63	1.95	9.49	7.54	3.20
BW2	Factorial	779	219.27	82.77	49.90	552.10	502.20	199.60
(mg)	full-sib	277	234.06	77.39	64.60	622.10	557.50	232.60
GR	Factorial	779	0.198	0.035	0.100	0.322	0.223	0.198
(mm/day)	full-sib	277	0.168	0.037	0.076	0.263	0.187	0.171

Age1: age at the start of metamorphosis, TL1: total length at the start of metamorphosis, PAL1: pre-anal length at the start of metamorphosis, BD1: body depth at the start of metamorphosis, Age2: age at the end of metamorphosis, TL2: total length at the end of metamorphosis, PAL2: pre-anal length at the end of metamorphosis, BD2: body depth at the end of metamorphosis, BW2: body weight at the end of metamorphosis, GR: growth rate calculated as TL2/Age2, ND: no data.

### Parentage assignment

Of the 810 offspring, 746 were successfully assigned to 86 of 114 possible families (S4 Table). The remaining 64 offspring could not be assigned unambiguously to a single parental pair because of insufficient resolution resulting from poor allelic amplification (Table 2).

**Table 2:** Number of progeny assigned to each of the families generated in the factorial cross.

	Dam												
Sire	1	2	3	4	5	6	7	8	9	10	11	Total	%
1	1	1	-	-	-	-	-	-	-	-	-	2	0.2
2	-	-	0	1	0	0	3	34	3	6	3	50	6.2
3	-	-	0	0	0	0	4	17	0	5	3	29	3.6
4	-	-	0	2	0	0	0	10	0	2	8	22	2.7
5	-	-	0	3	0	0	13	25	3	11	11	66	8.1
6	2	12	3	2	5	4	9	20	1	3	10	71	8.8
7	2	1	-	-	-	-	-	-	-	-	-	3	0.4
8	3	4	-	-	-	-	-	-	-	-	-	7	0.9
9	1	0	1	0	0	4	0	7	7	8	6	34	4.2
10	3	4	0	1	2	0	0	21	0	3	1	35	4.3
11	3	5	10	16	0	0	6	51	10	13	16	130	16.0
12	4	14	1	4	37	34	3	22	2	19	28	168	20.7
13	1	11	1	0	15	3	-	-	-	-	-	31	3.8
14	3	19	4	3	4	3	0	20	11	8	23	98	12.1
unknown	0	6	2	5	5	4	0	22	1	10	9	64	7.9
Total	23	77	22	37	68	52	38	249	38	88	118	810	-
%	2.8	9.5	2.7	4.6	8.4	6.4	4.7	30.7	4.7	10.9	14.6	-	100

"-" indicates that mating was not performed.

### Trait heritability and correlations

The heritabilities and genetic and phenotypic correlations among the studied traits are shown in Table 3. Regarding the former, the estimated heritabilities were 0.41 and 0.36 for the timing of metamorphosis (Age1 and Age2, respectively) and 0.20 and 0.32 for the weight and growth rate of glass eels (BW2 and GR), and ranged from 0.16 to 0.33 for morphometric traits (TL, PAL, BD). As expected, strong genetic correlations were observed for timing and morphometric traits between the start and end of metamorphosis (1.00, 0.97, 0.89 for Age, TL, PAL, respectively), with the exception of body depth (0.29). The morphometric traits and weights of glass eels mainly correlated positively with the timing of metamorphosis, with low values for phenotypic correlations and medium values for genetic correlations. The genetic correlations of growth rate with the timing of metamorphosis were negative and strong, whereas those with the body size at metamorphosis were generally weak.

**Table 3:** Heritabilities (bold, diagonal values with standard errors), genetic correlations (below diagonal values), and phenotypic correlations (above the diagonal) among metamorphic traits in captive-bred Japanese eel.

Traits	Age1	TL1	PAL1	BD1	Age2	TL2	PAL2	BD2	BW2	GR
Age1	**0.41 ± 0.22**	0.27	0.23	0.15	0.88	0.24	0.31	0.34	0.45	-0.74
TL1	0.52	**0.33 ± 0.16**	0.77	0.48	0.29	0.65	0.53	0.52	0.70	0.10
PAL1	0.63	0.99	**0.24 ± 0.12**	0.64	0.22	0.45	0.37	0.43	0.57	0.03
BD1	0.19	0.37	0.30	**0.16 ± 0.10**	0.17	0.35	0.26	0.47	0.51	0.04
Age2	1.00	0.54	0.64	0.22	**0.36 ± 0.20**	0.51	0.53	0.51	0.52	-0.55
TL2	0.53	0.97	0.95	0.46	0.55	**0.23 ± 0.13**	0.82	0.66	0.68	0.37
PAL2	0.37	0.89	0.89	-0.06	0.37	0.83	**0.18 ± 0.11**	0.64	0.67	0.20
BD2	0.69	0.85	0.88	0.29	0.69	0.80	0.73	**0.16 ± 0.11**	0.88	0.06
BW2	0.63	0.89	0.90	0.39	0.63	0.86	0.74	0.98	**0.20 ± 0.12**	-0.04
GR	-0.90	-0.11	-0.25	0.02	-0.89	-0.11	0.00	-0.41	-0.31	**0.32 ± 0.17**

### The ddRAD-seq and linkage map

SNP genotype data was obtained from 240 samples of full-sib family including 238 offspring and their parents. A total of 1,262 SNP marker loci, which included 386 (30.6%) “nn x np” type markers, 401 loci (31.8%) “lm x ll” type markers, 275 loci (21.8%) “ef x eg” type markers and 200 loci (15.8%) “ab x cd” type markers, were used for linkage mapping after filtration of genotyping quality and SNPs calling rate (> 90%) (S5 table).

Both male and female maps comprised 19 linkage groups (LGs), which corresponds with the karyotypes of Japanese eel (2n = 38), and contained 851 and 861 SNP loci, respectively (Table 4). The male map spanned a length of 1432.9 cM, and the length of each LG ranged from 49.9 to 113.6 cM (mean 75.4 cM). The number of markers per LG varied from 24 to 73, with an average of 44.8 markers per LG, and the average distance spacing between two markers was 3.48 cM. The female map spanned a length of 1884.1 cM, and the length of each LG ranged from 47.8 to 148.2 cM (mean 99.2 cM). The number of markers per LG varied from 18 to 72, with an average of 45.6 markers per linkage group, and the average distance spacing between two markers was 3.39 cM.

**Table 4:** Number of SNP markers, genetic length for each linkage group.

Linkage group	Male map		Female map		Consensus map	
	No. of SNP markers	Length (cM)	No. of SNP markers	Length (cM)	No. of SNP markers	Length (cM)
LG1	50	107.0	49	141.6	73	124.3
LG2	57	106.3	57	129.4	79	118.1
LG3	42	60.2	36	84.6	59	72.4
LG4	53	62.9	51	106.7	75	86.6
LG5	52	74.7	51	130.6	78	104.0
LG6	45	69.4	44	95.4	62	97.2
LG7	73	113.6	67	148.2	101	135.6
LG8	52	102.6	52	124.9	75	113.7
LG9	49	77.5	52	109.9	77	95.3
LG10	33	49.9	32	67.5	43	59.8
LG11	36	55.6	40	77.1	56	70.8
LG12	40	50.1	44	85.1	58	68.0
LG13	34	57.3	36	86.3	51	80.3
LG14	28	59.5	30	57.0	45	59.5
LG15	54	76.6	72	109.2	88	92.9
LG16	36	91.3	44	92.9	57	92.1
LG17	24	58.3	18	47.8	30	62.7
LG18	42	53.5	48	74.7	67	64.8
LG19	51	106.4	43	115.4	68	110.9
Total	851	1432.9	866	1884.1	1242	1709.0

**Fig 2:**
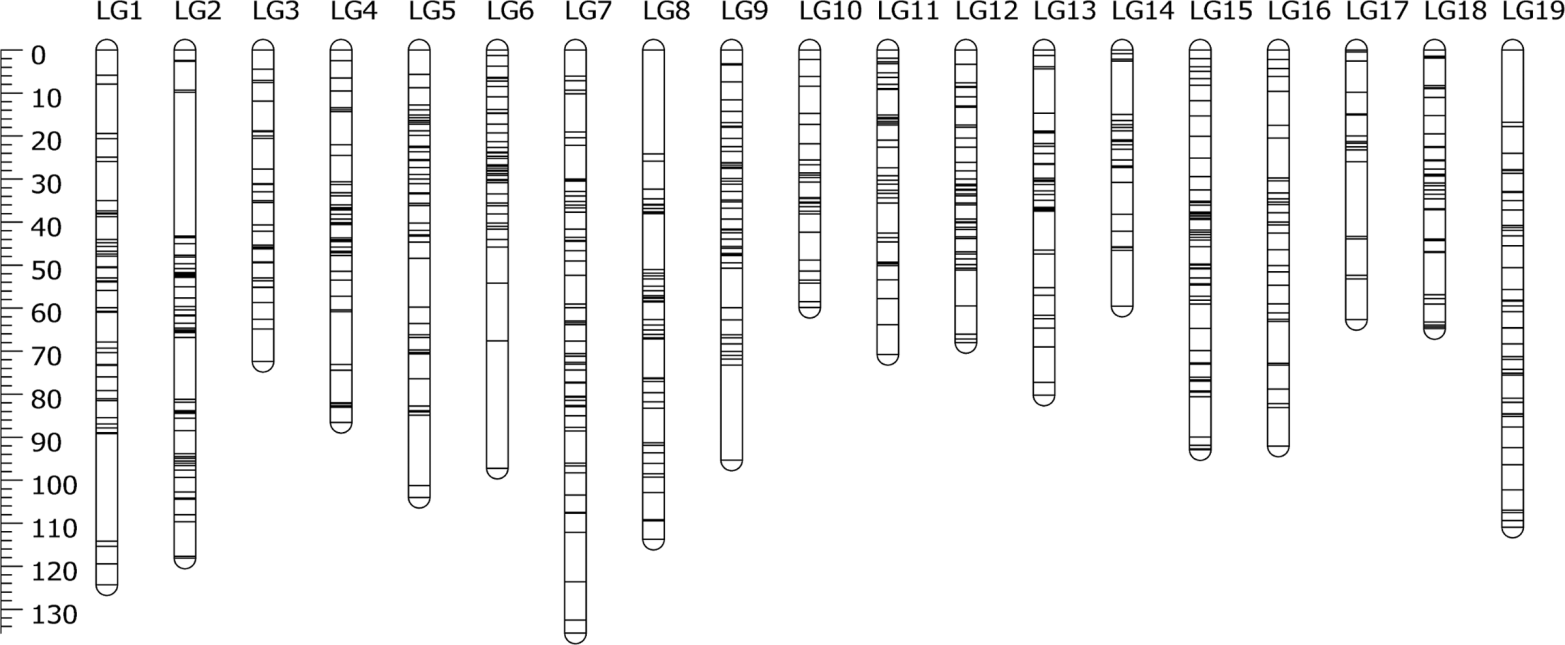
The consensus linkage map of Japanese eel.

Both sex-specific linkage maps were integrated into a consensus map based on the 475 shared SNP markers (Fig 2). As summarized in Table 4, the consensus map comprised 19 LGs, and contained 1,242 SNP loci. The map spanned a length of 1709.0 cM, and the length of each LG ranged from 59.5 to 135.6 cM (mean 89.9 cM). The number of markers per LG varied from 30 to 101, with an average of 65.4 markers per LG, and the average distance spacing between two markers was 2.27 cM.

**Fig 3:**
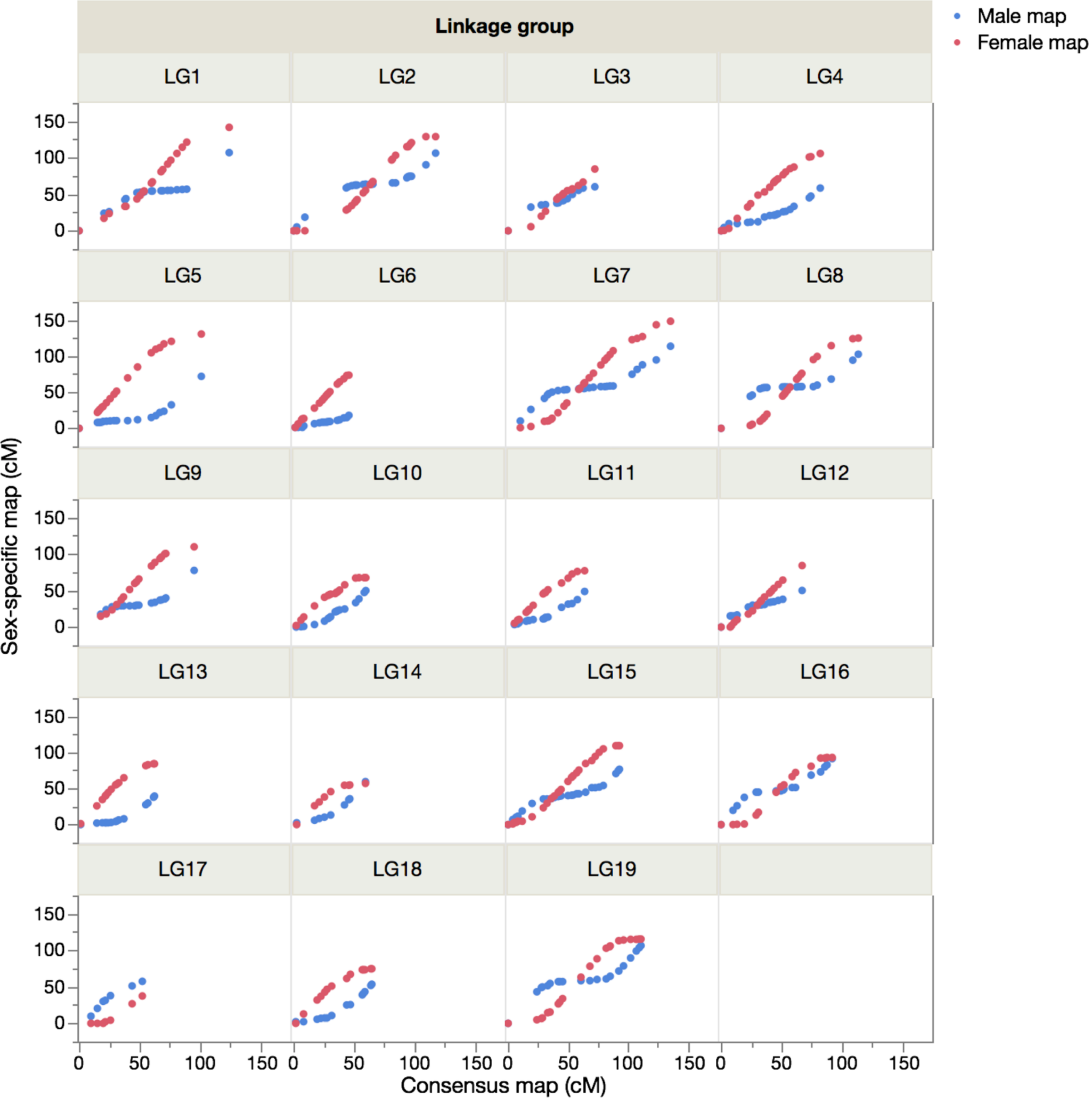
Comparisons of the recombination rates of shared markers in each linkage group between the consensus and sex-specific maps of Japanese eels.

The total genetic distances of the male, female and consensus maps were 1,432.9, 1,884.1 and 1,709.0 cM, respectively. Using the method of Chakravarti et al[29], the corrected lengths of the maps were estimated at 1,575.0, 2,028.8, and 1,799.4 cM, which were converted into genome coverages of 85.6, 83.9 and 85.0%, respectively. The total genetic distances within maps differed significantly by sex: the female-to-male LG length ratios ranged from 0.82 (LG17) to 1.75 (LG5) (average: 1.33), and the female-to-male inter-locus distance ratios ranged from 0.66 (LG18) to 1.66 (LG10) (average: 1.06). In Fig 3, we have illustrated the genetic distance relationship between the consensus and sex-specific maps to visualize the sex-based differences in recombination rates for 475 shared SNP markers.

### QTL analysis

**Fig 4:**
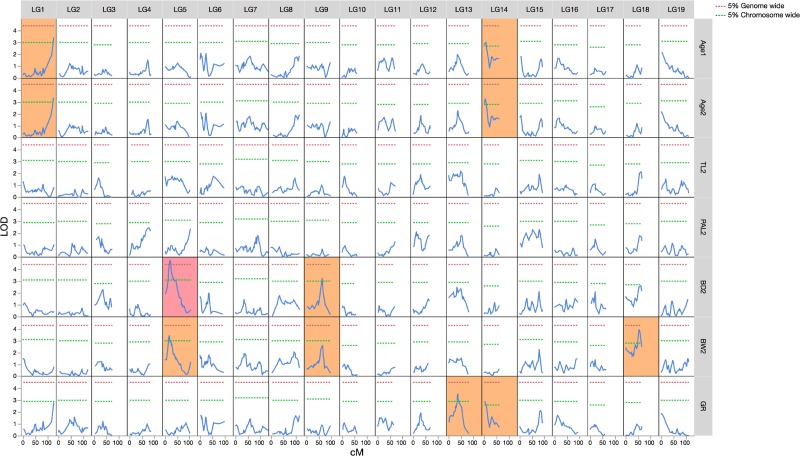
Simple interval mapping of QTLs for metamorphic traits in Japanese eels.

Simple interval mapping was used to detect one significant QTL (genome-wide *P* < 0.05) and five suggestive QTLs (chromosome-wide *P* < 0.05) associated with metamorphic traits (Fig 4). For the timing of metamorphosis (Age1 and Age2), two suggestive QTLs that explained 8.4% and 7.8% of the total phenotypic variance in Age2 were detected on LG1 and LG14, respectively. Regarding morphometric traits at the end of metamorphosis, one significant QTL and one suggestive QTL for BD2 that explained 11.8% and 7.6% of the total phenotypic variance were identified on LG5 and LG9, respectively. However, no QTLs were identified for TL2 or PAL2. Although the QTL profile for BW2 was quite similar to that for BD2 (*r* = 0.817), we also found three suggestive QTLs that explained 9.2%, 8.6%, and 7.7% of the total phenotypic variance in this parameter on LG5, LG9, and LG18, respectively. The QTL profile for GR was quite similar to the profiles for Age1 and Age2 (*r* = 0.766 and 0.758, respectively), although two suggestive QTLs that explained 8.3% and 6.8% of the total phenotypic variance were detected on LG13 and LG14, respectively.

### Draft genome sequence and genetic map integration

From two haploid Japanese eel larvae, we obtained 85-Gb (77x) reads using the single-end method (insert size: 200 bp) and Ion Proton sequencer, and 75 Gb (68x) reads using the paired-end method (insert size: 240–480 bp) and Next Seq 500 sequencer. We also obtained 73-Gb (66x) reads from the dam using the paired-end method (insert size: 720 bp) and mate-pair method (insert size: 2–20 kb) and Next Seq 500 sequencer. After scaffolding, the total genome measured 939,158,716 bp and contained 2,304 scaffolds (Table 5). Contig N50 comprised 3,016,897 bp, and scaffold N50 comprised 3,042,577 bp.

**Table 5:** Summary of the assembly statistics of Japanese eels.

*Contig statistics (≥ 500 bp)*	
Number of contigs	18,358
Average contig length (bp)	51,909
N50 contig length (bp)	3,016,897
Largest contig length (bp)	20,113,222
*Scaffold statistics (≥ 2 kb)*	
Number of scaffolds	2,304
Average scaffold length (bp)	407,621
N50 scaffold length (bp)	3,042,577
Largest scaffold length (bp)	20,113,222
*Scaffold statistics (≥ 2 kb) recostructed by ALLMAPS*	
Number of scaffolds	2,741
Average scaffold length (bp)	342,631
N50 scaffold length (bp)	1,596,562
Largest scaffold length (bp)	11,243,566

**Fig 5:**
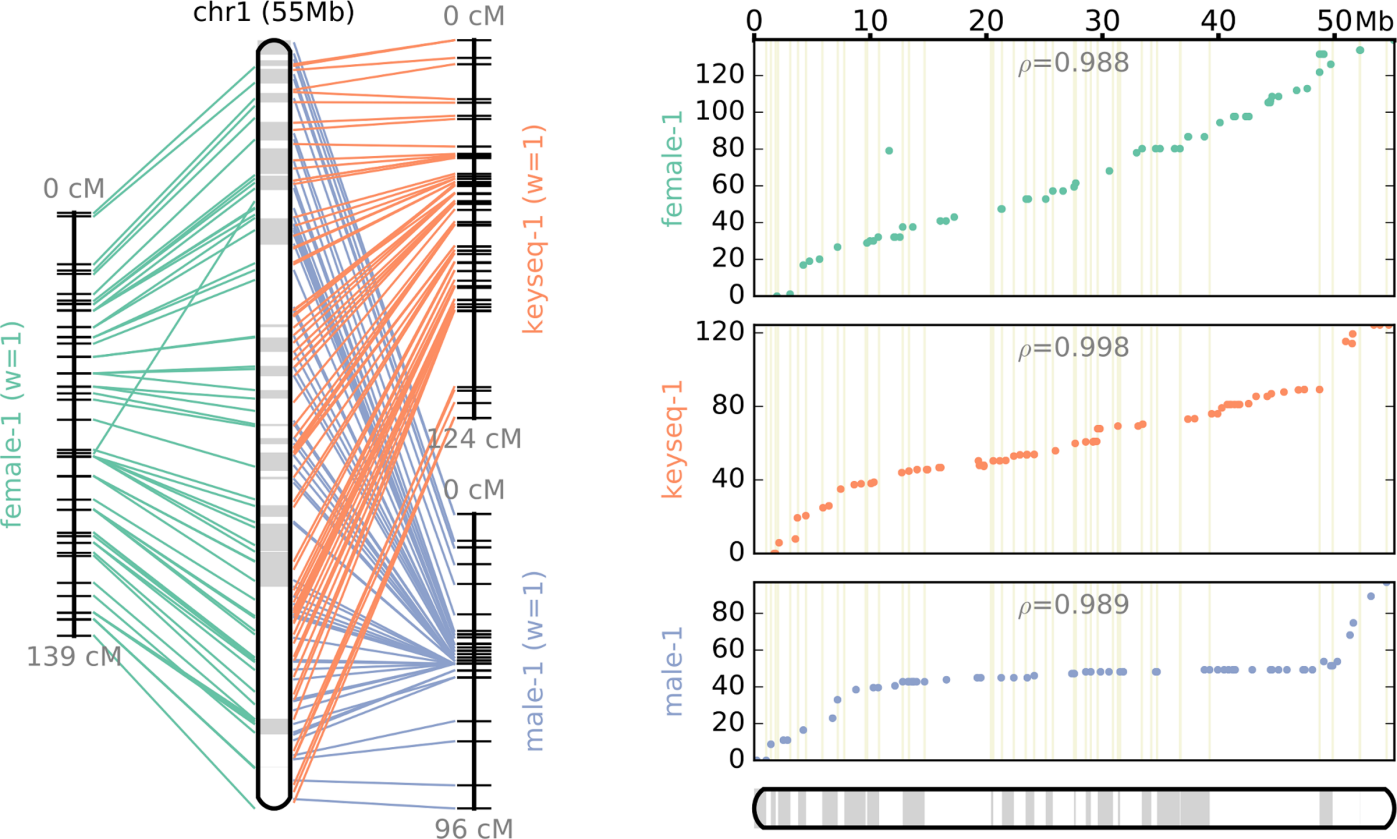
Pseudochromosome 1 of the Japanese eel genome, reconstructed from three input maps by ALLMAPS. **“**Female-1” and “Male-1” indicate the LG1 from the female and male genetic linkage maps published by Kai et al. (2014). “Keyseq-1” indicates the LG1 of the consensus genetic linkage map developed in the present study.

We next targeted markers to specific scaffold regions using ALLMAPS software, which allowed us to identify the chromosomal order of the scaffolds in our constructed Japanese eel genome assembly. We used three genetic maps (two sex-specific maps by Kai et al., 2014[9] and a newly constructed sex-average map) simultaneously to anchor 746 Mb (77.2% of the current assembly) onto 19 chromosomes (Fig 5 and S1 File and S6 and S7 Tables). Reconstruction of the genome assembly yielded a total genome of 939,152,857 bp, containing 2,741 scaffolds. Additionally, the N50 scaffold lengths decreased drastically from 3,042,577 to 1,596,562 bp (Table 5). The concordance between the final reconstructed assembly and each of the three maps matched the assigned equal weights. The mean of Spearman’s correlation coefficient of all linkage groups of the male, female and consensus maps were 0.982, 0.982 and 0.980, respectively.

Final genome completeness percentages of 96.77% and 93.93% were estimated by CEGMA and BUSCO, respectively (S8 Table).

## Discussion

This paper highlights several important genetic aspects of traits exhibited during the metamorphosis of *A*. *japonica* from leptocephali into glass eels. To our best knowledge, this is the first published report of the heritability and genetic architecture of traits related to body size and timing at metamorphosis in captive-bred Japanese eels. In addition, we also updated the ddRAD-based consensus linkage map and draft genome and generated a high-quality reference sequence using a genetic linkage map and an integrated approach to *de novo* genome assembly.

The timing of metamorphosis from leptocephali to glass eels is an ecologically and evolutionarily important trait in the natural environment[36]. As oceanic currents transport leptocephali over long distances from the spawning area, the timing of metamorphosis can greatly affect the ultimate destinations of eels. Otolith microstructure analyses of wild glass eels have evaluated differences in the timing of metamorphosis among freshwater eel species, as well as the growth rates of leptocephali and the body size at metamorphosis[16,17,37]. Previous studies of anguillid eels suggest that the evolutionary history may be reflected by differences in early life parameters, such as a smaller maximum larval size and more rapid growth among tropical species with short migrations relative to temperate species with longer migrations [38], as anguillids first evolved as tropical species from mesopelagic eels[39,40]. However, the challenge of larval rearing in a laboratory setting has inhibited quantitative genetic studies of artificially propagated families. Our present results from the estimation of genetic parameters proved that traits related to body size and the timing at metamorphosis were moderately heritable and exhibited weakly positive correlations with each other. The information may also facilitate an understanding of the mechanism of metamorphosis from leptocephali to glass eels while clarifying details of the ecology, larval transportation strategies evolution, and speciation processes of freshwater eels.

The observed heritability of traits associated with body size and the timing of metamorphosis suggest that an effective selection program intended to improve both traits in Japanese eel may encompass considerable genetic variation. Mass selection by simple truncation to shorten the larval period may be paralleled by body size miniaturization at metamorphosis, and an appropriate weighted selection index will be required if this trait is undesired by breeders. In such cases, the growth rate of the whole larval period (GR) may be a good indicator. However, we note that the limited numbers of parental fishes (14 sires and 11 dams) and small, uneven family sizes in our study could result in the under- or overestimation of the heritability of each trait. In particular, three of the 11 dams produced 56.2% (455/810) of the offspring and three of the 14 sires produced 48.9% (396/810) of the offspring, and the intersection of these six individuals produced 26.8% (200/746) of the total assignable offspring (Table 2). These unbalanced sample sizes may have likely had a measurable impact on the overall findings in our study, although the estimation of the variance components as random effects using maximum likelihood (ML) or restricted ML (REML) does not produce negative variance components and is not sensitive to unbalanced sample sizes. In addition, heritability may have also been biased by common environmental effects and confounding maternal effects. Accordingly, heritabilities, and particularly genetic correlations, must be interpreted carefully.

In our previous study, we used the double pseudo-test cross method[41] to construct sex-specific (female and male) maps using an F_1_ family (parents and 92 offspring) as a mapping panel. Despite a sufficiently large number of markers (2,672 SNPs and 115 SSRs), we were unable to construct a consensus map that integrated the sex-specific maps, and our genetic maps contained several large gaps. Therefore, in the present study, we used ddRAD sequencing to genotype a larger number of offspring (238 individuals), and used the multipoint maximum likelihood mapping algorithm and the population type of CP in JONMAP 4.1 to construct a consensus map. Although we successfully constructed a consensus genetic linkage map, it contained fewer mapped SNP markers (1,242 SNPs) than the previous map; in addition, the map resolution improved only slightly, and the large gaps between markers remained. The total lengths of the present genetic maps (female: 1884.1 cM, male: 1432.9 cM, consensus: 1709.0 cM) were slightly longer than those of previous maps (female: 1748.8 cM, male: 1294.6 cM), while the map lengths and female-to-male ratio of each LG were highly similar between the previous and present maps, except for the LG3 of male maps (previous map: 19.6 cM, present map: 60.2 cM).

The production of high-quality genome assemblies relies on the use of a range of mapping information, including high-density genetic linkage maps [33,42]. In the previous study, we integrated a genetic linkage map with the publicly available draft genome sequence of Japanese eels [26]. However, we were unable to identify the orientations of most scaffolds on the LGs because of the incomplete and highly fragmented nature of the draft genome, and we rarely observed genetically separated markers within the scaffolds. Therefore, in the present study, we integrated a newly developed *de novo* genome assembly and genetic linkage map of the Japanese eel using ALLMAPS software, and successfully anchored 746 Mb (77.2%) of newly assembled sequence onto the 19 LGs. This represented a significant improvement of the genome assembly over a previous draft based on short reads only, both in terms of contiguity and structural quality. In our genome, although the N50 scaffold lengths drastically decreased because of splitting the chimeric scaffolds prior to the execution of ALLMAPS, the scaffold N50 (1.6 Mb) represents a substantial improvement over the previously established genomic resources for *A*. *japonica* (52.8 kb: Henkel et al., 2012a [26]), *A*. *rostrata* (86.6 kb: Pavey et al., 2016 [43]), and *A*. *anguilla* (77.8 kb: Henkel et al., 2012b [44]; 1.2 Mb: Jansen et al., 2017 [45]). The completeness of the genome assembly was evaluated against a set of 248 core eukaryotic genes by CEGMA, and 240 (96.77%) genes of these subset were completely covered in our genome (S8 Table). These metrics offer substantial improvement over the previously established genomic resources for *A*. *japonica* (85.9%: Henkel et al., 2012a [26]), *A*. *rostrata* (89.1%: Pavey et al., 2016 [43]), and *A*. *anguilla* (76.1%: Henkel et al., 2012b [44]). Accordingly, we are accumulating genomic information to construct a comprehensive genome database based on this Japanese eel draft genome.

One of the main reasons for mapping QTL was to use mapped QTL for selection purposes in marker-assisted selection (MAS). Our QTL analysis identified one significant QTL (genome-wide *P* < 0.05) and five suggestive QTLs (chromosome-wide *P* < 0.05) related to body size and the timing at metamorphosis, suggesting that these traits exhibit a polygenic genetic structure comprising many QTLs with small effects. Although we must consider that wild population may contain QTLs with large effects that did not separate in our studied family, the success of MAS schemes might be very limited because the explained variance by the mapped QTLs were very small. Accordingly, it would be appropriate to apply breeding schemes, such as family-based selective breeding, best linear unbiased prediction (BLUP), and genomic selection (GS), rather than MAS of specific QTLs to the genetic improvement of these traits. Compared with MAS, GS can more effectively use genome-wide genotype datasets with higher densities, and is therefore likely to become the gold standard of selective breeding in the near future[46]. On the other hand, the ultimate goal of a QTL mapping experiment is to detect the underlying gene and the causative mutation within the gene. Although several QTLs were mapped in this study, the mapping precision was very low and it was impossible to identify the gene region. In order to identify the underlying gene, it will be necessary to apply large-scale genome-wide association studies (GWAS). GWAS results will also provide more detailed information on the genetic architecture of these quantitative traits, and the information will improve genomic selection. Combined with improved reference genome and high-density SNP genotyping, it will be possible to apply GWAS and genomic selection for Japanese eel in near future.

## Conclusion

Our findings suggest for the first time that for captive-bred Japanese eels, traits associated with body size and the timing of metamorphosis from leptocephali to glass eels were heritable and intercorrelated. The results of our QTL analysis suggest that these traits exhibit a polygenic genetic structure comprising many QTLs with small effects. In addition, we have integrated our updated genetic linkage map and newly generated *de novo* genome assembly for the Japanese eel. We expect that the information and tools reported herein will further the development of freshwater eel genetics and genomics.

## Supporting information

S1 Table List of the short tandem repeat (STR) markers used for parentage assignment. (XLSX)

S2 Table Next-generation sequencing (NGS) information. (XLSX)

S3 Table Phenotype data used for quantitative trait locus (QTL) mapping. (XLSX)

S4 Table Phenotype and pedigree data for the estimation of genetic parameters by ASReml. (XLSX)

S5 Table Single nucleotide polymorphism (SNP) genotype data used for linkage mapping. (XLSX)

S6 Table Summary statistics of the integration for each of the three component maps of the Japanese eel (female map, male map, consensus map with equal weights) and the final consensus anchoring (“anchored”). (XLSX)

S7 Table List of SNP markers and anchored draft genome. (XLSX)

S8 Table Completeness assessment results by CEGMA and BUSCO. (XLSX)

S1 File Pseudochromosome 1–19 from the Japanese eel genome, reconstructed from three input maps by ALLMAPS. (PDF)
