# Supplementary figures and images for: Genetic parameters and quantitative trait loci analysis associated with body size and timing at metamorphosis into glass eels in captive-bred Japanese eels (Anguilla japonica)
Source: PLoS One. 2018 Aug 29;13(8):e0201784. doi: 10.1371/journal.pone.0201784 (PMC6114518; doi:10.1371/journal.pone.0201784)

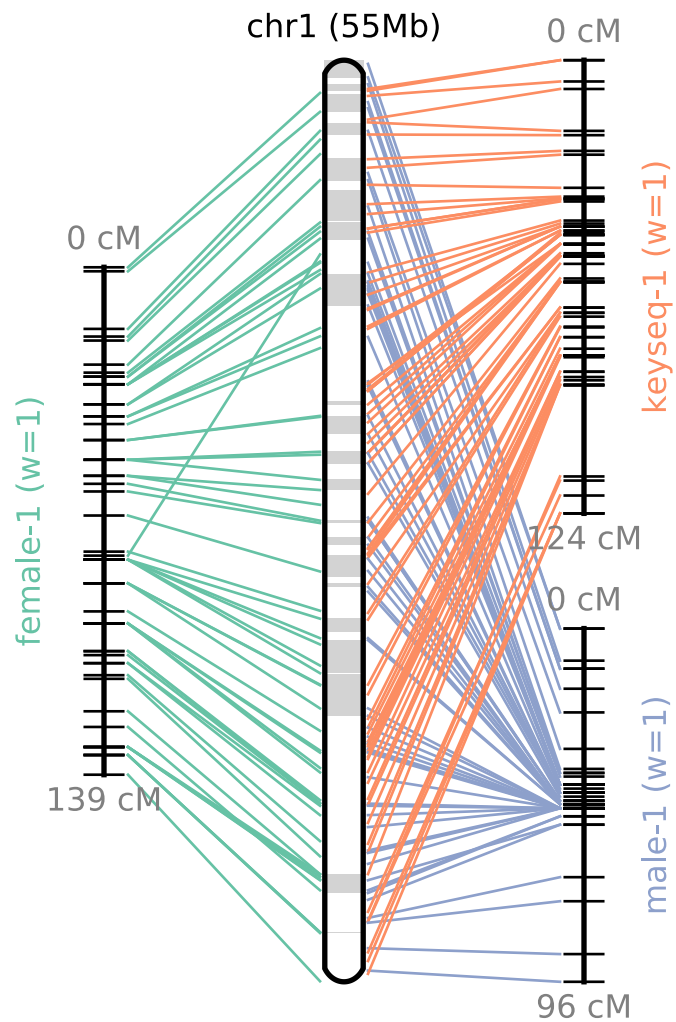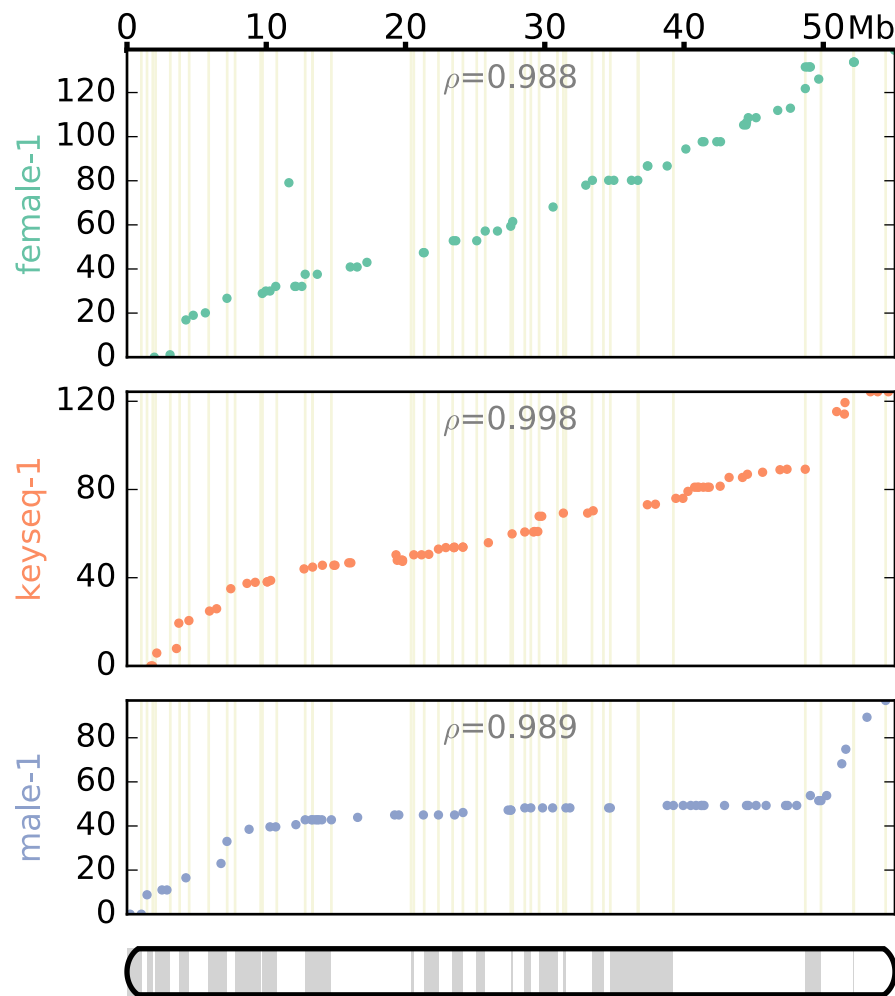

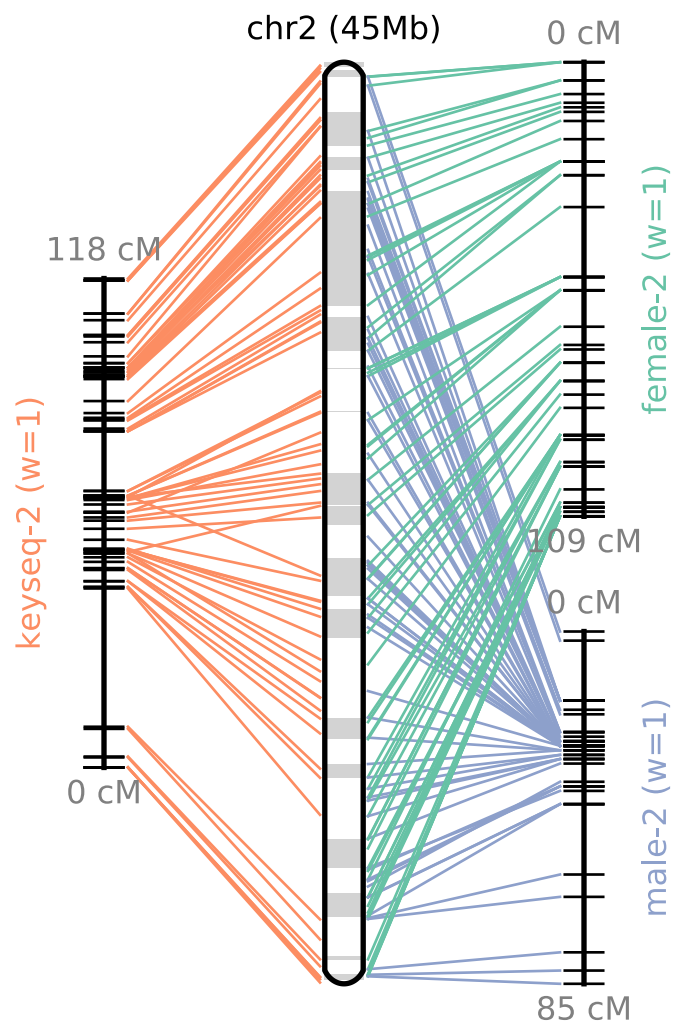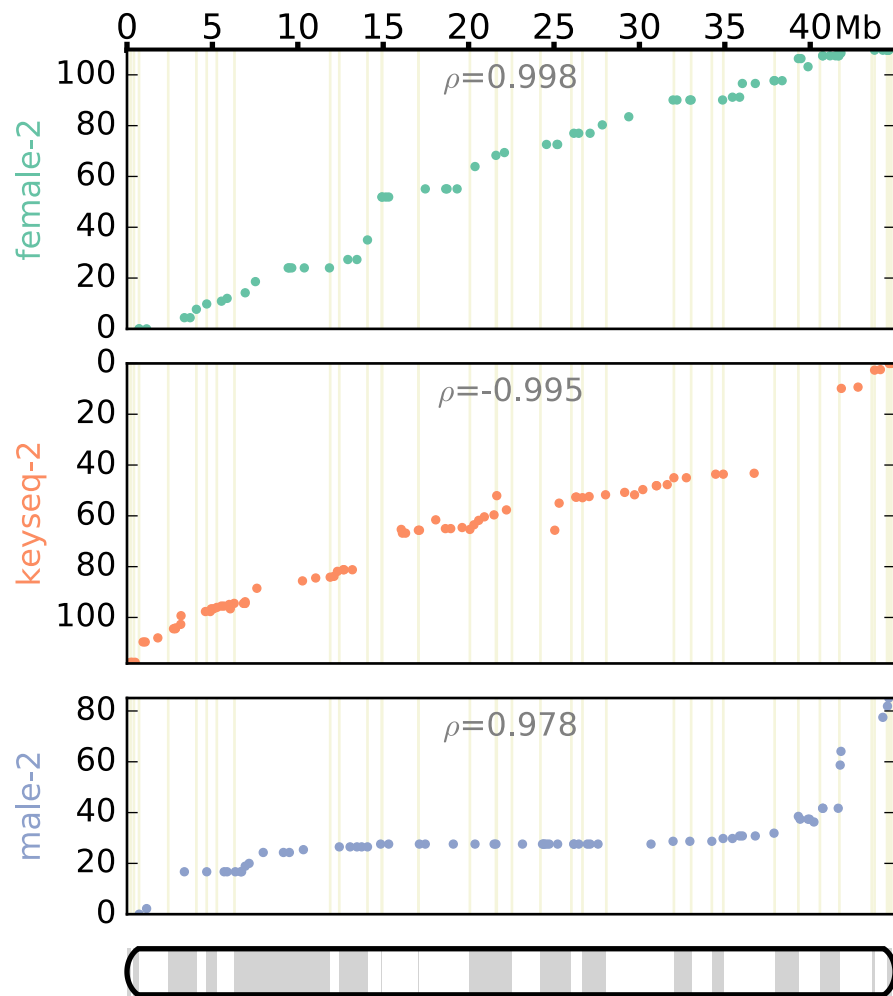

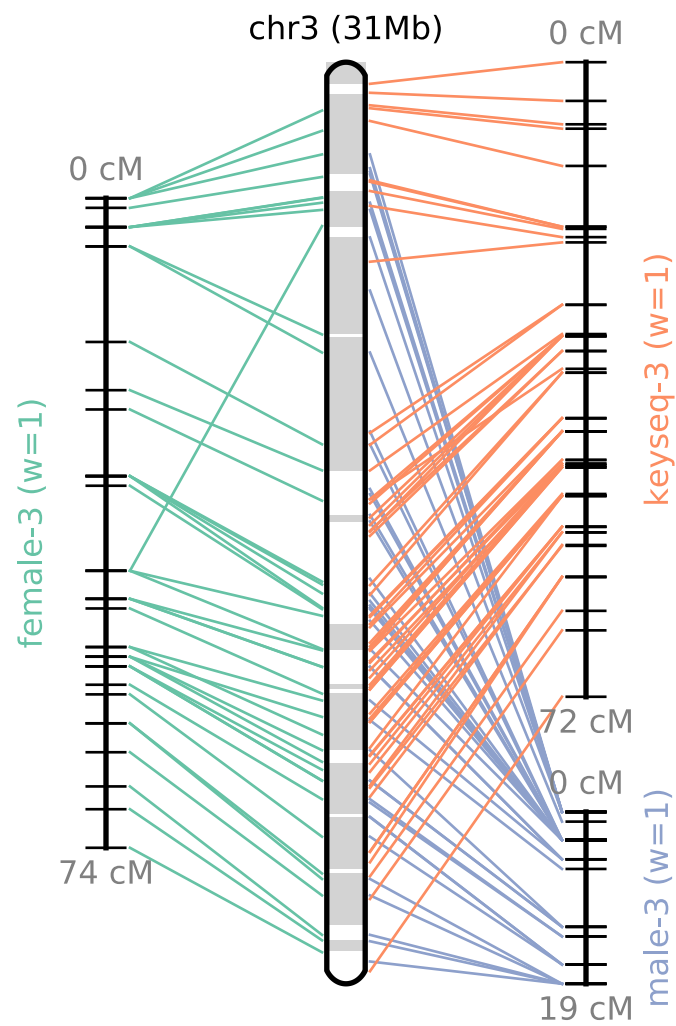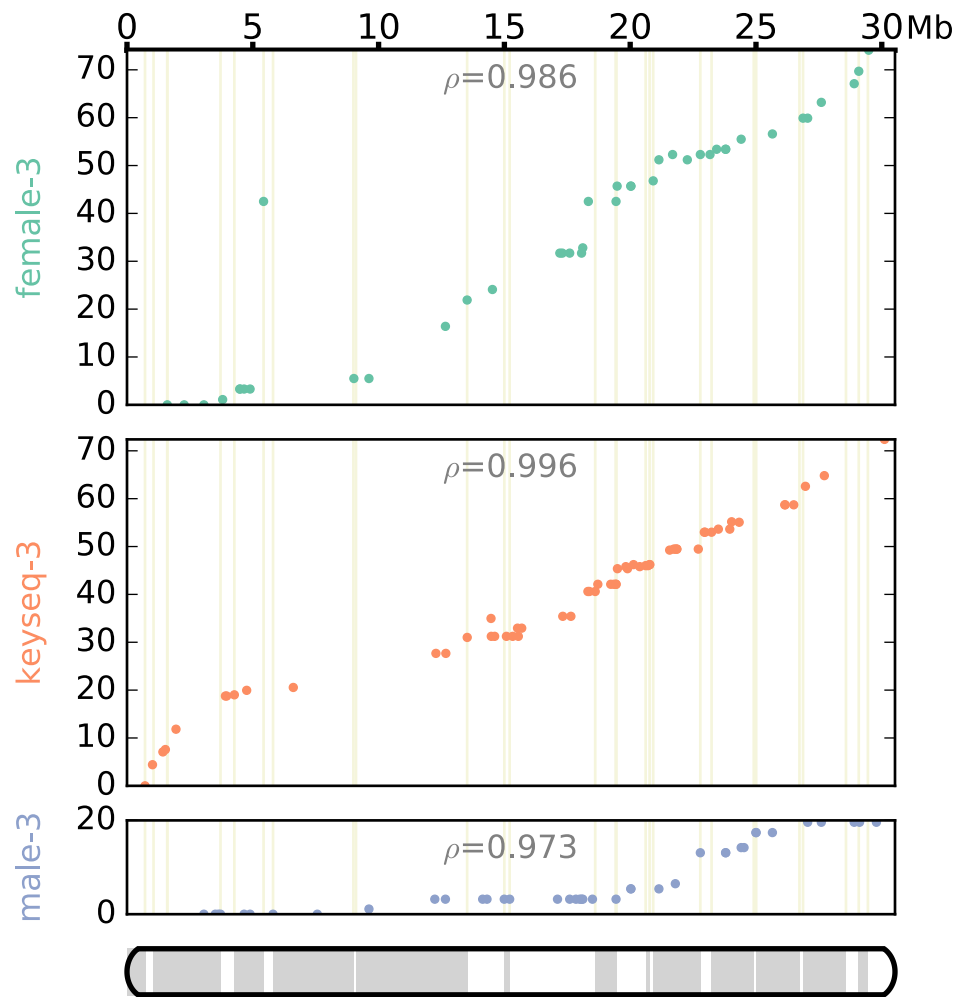

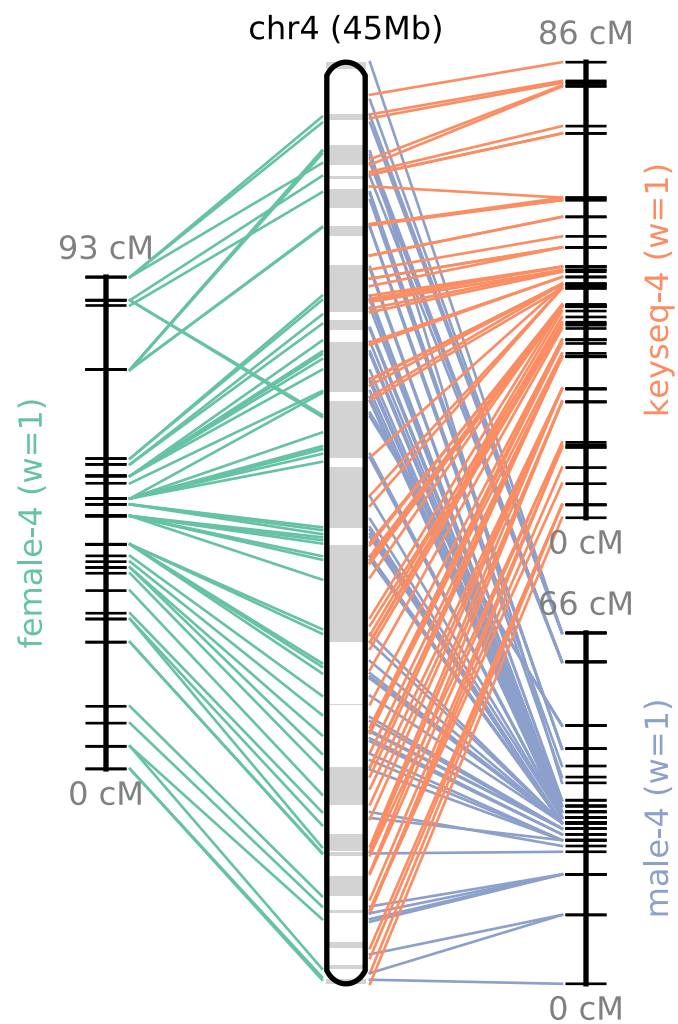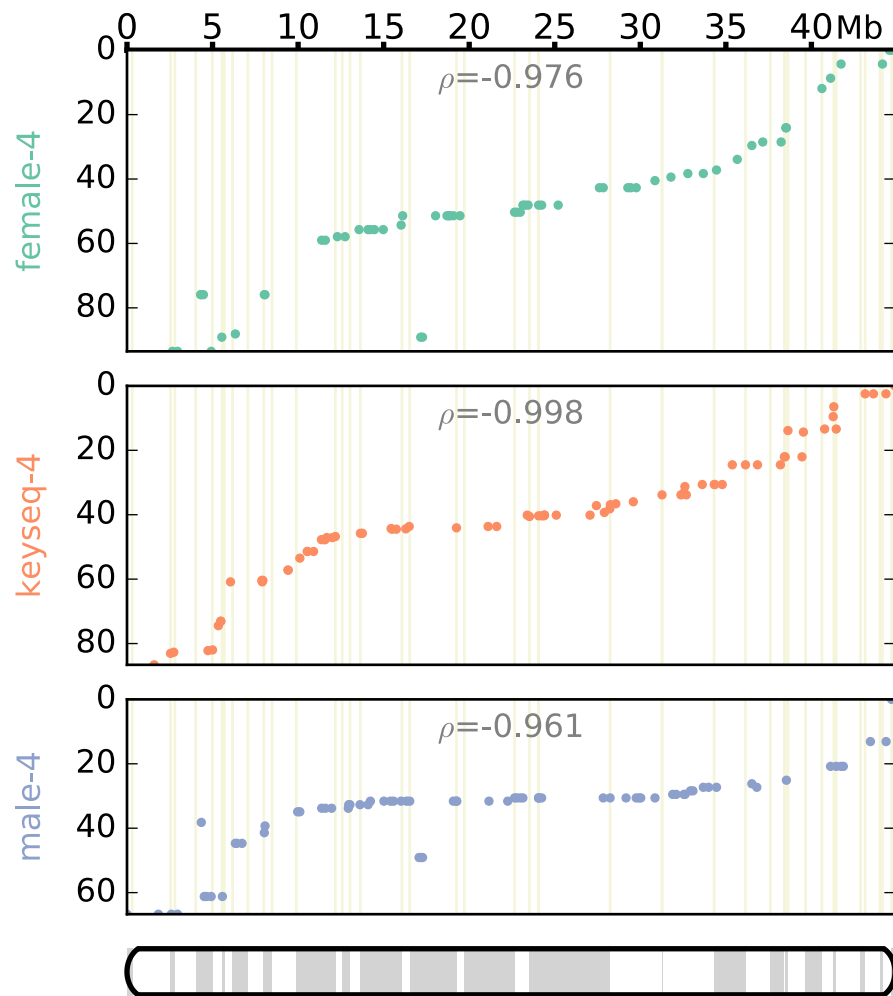

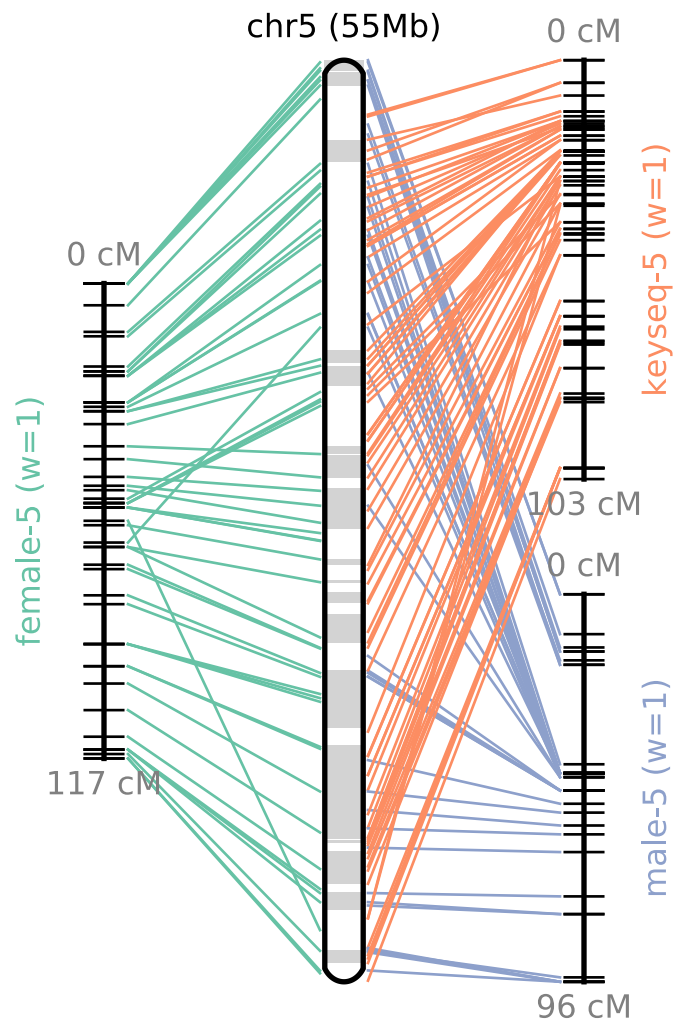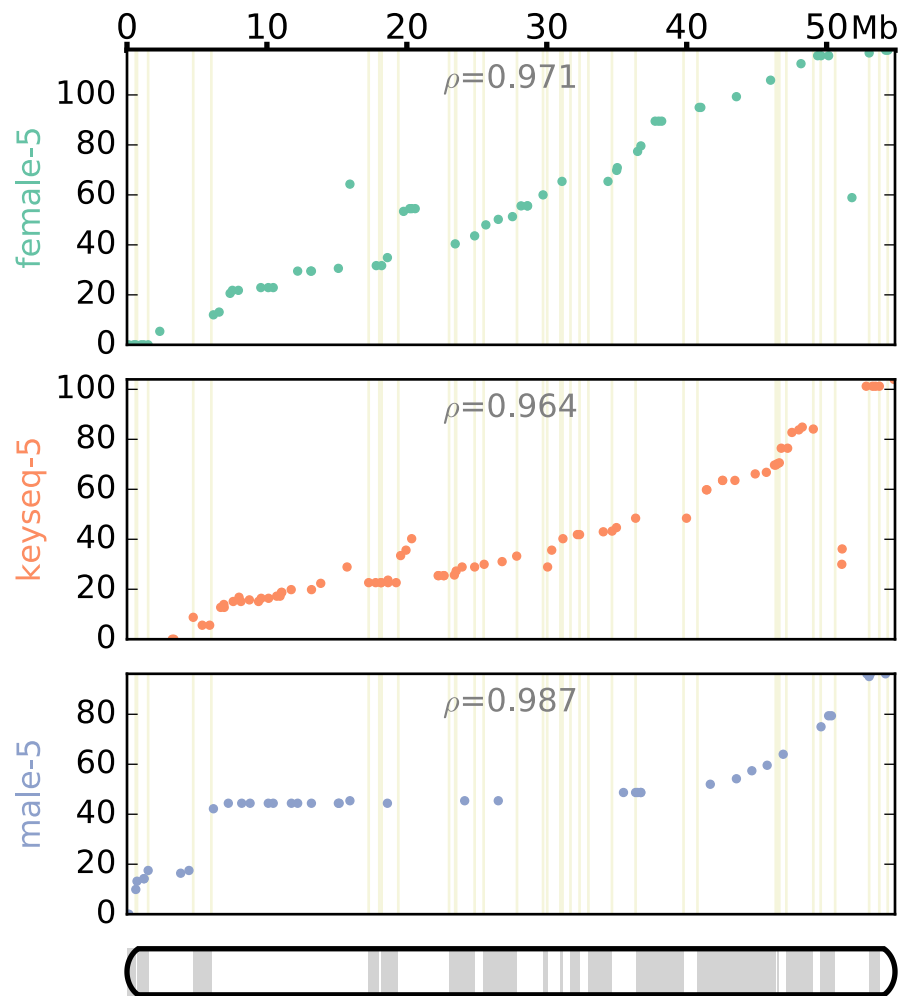

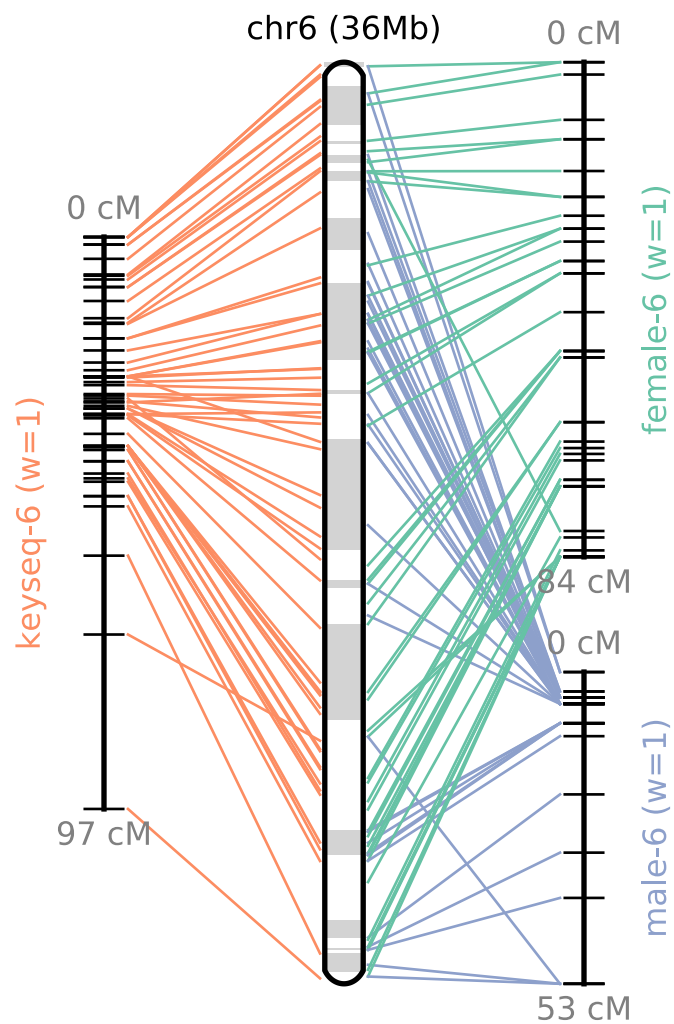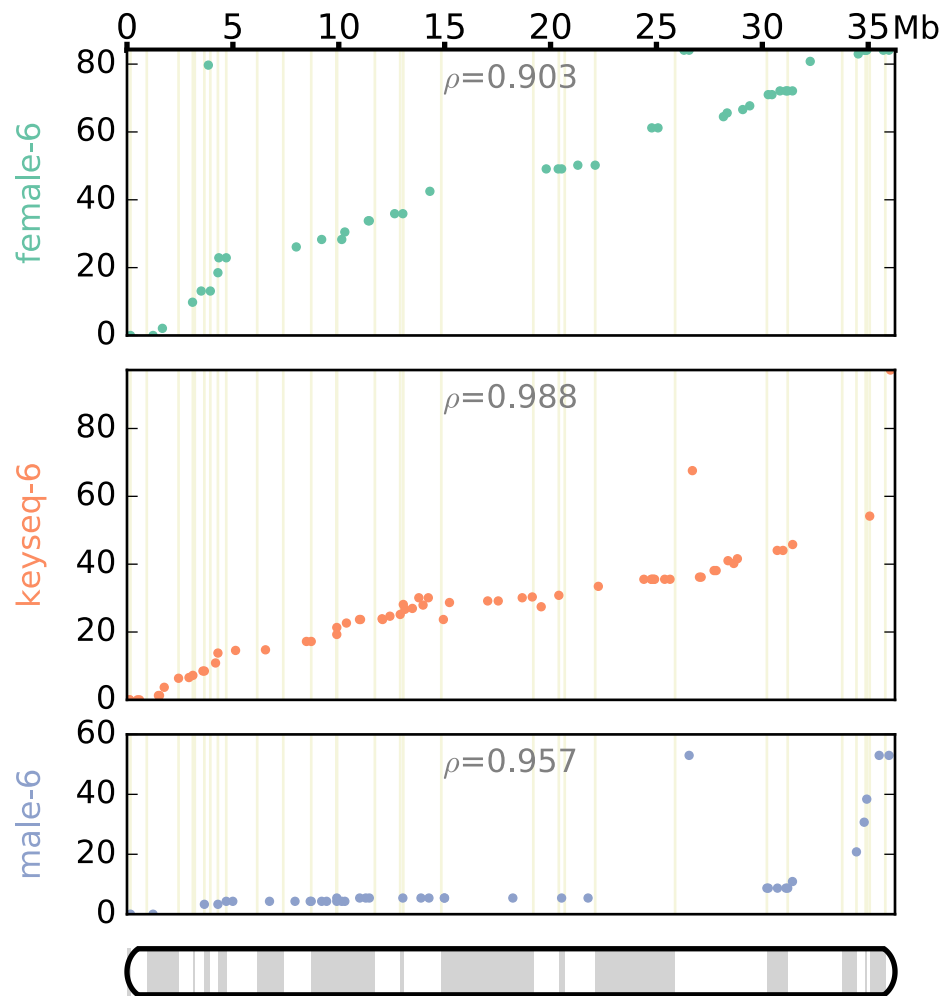

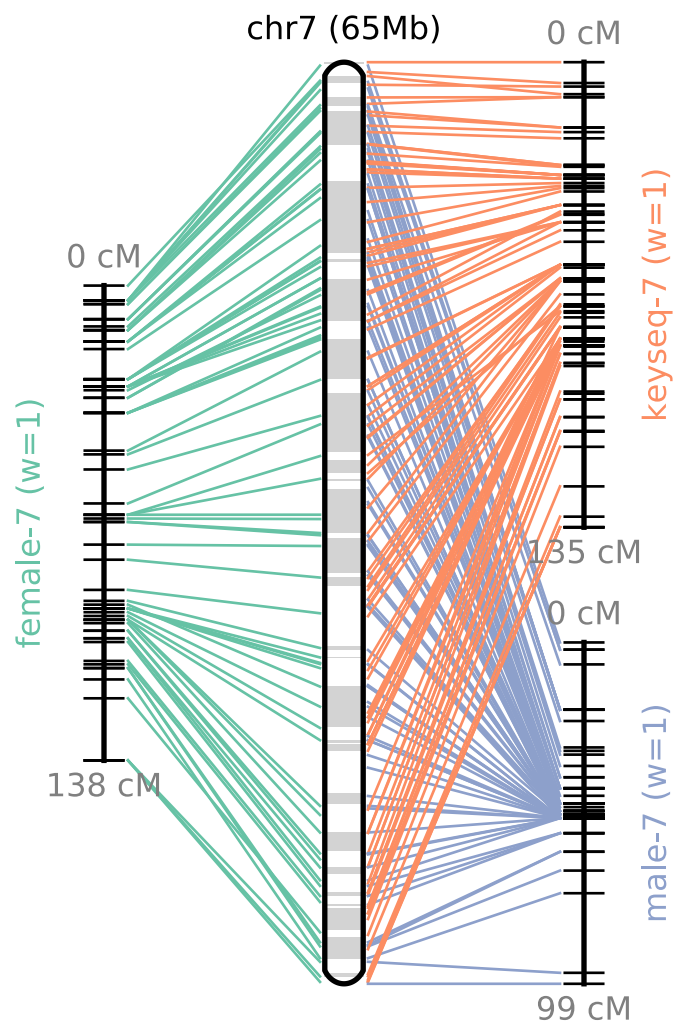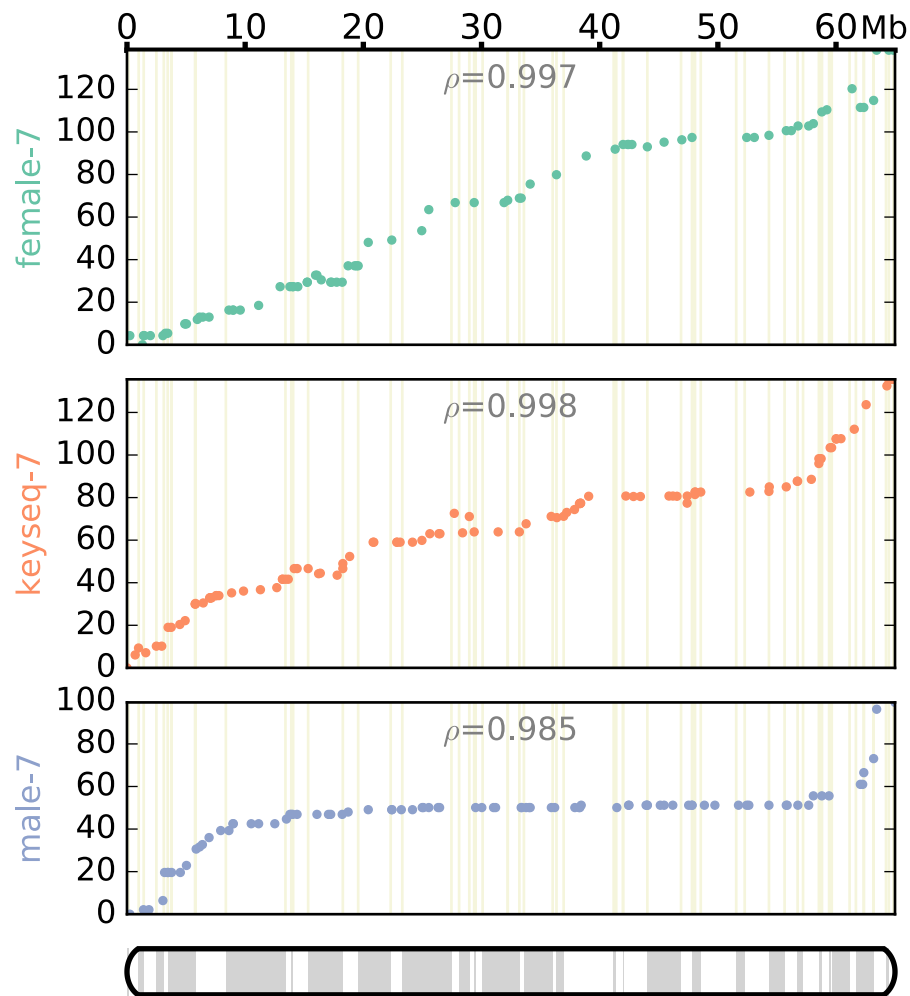

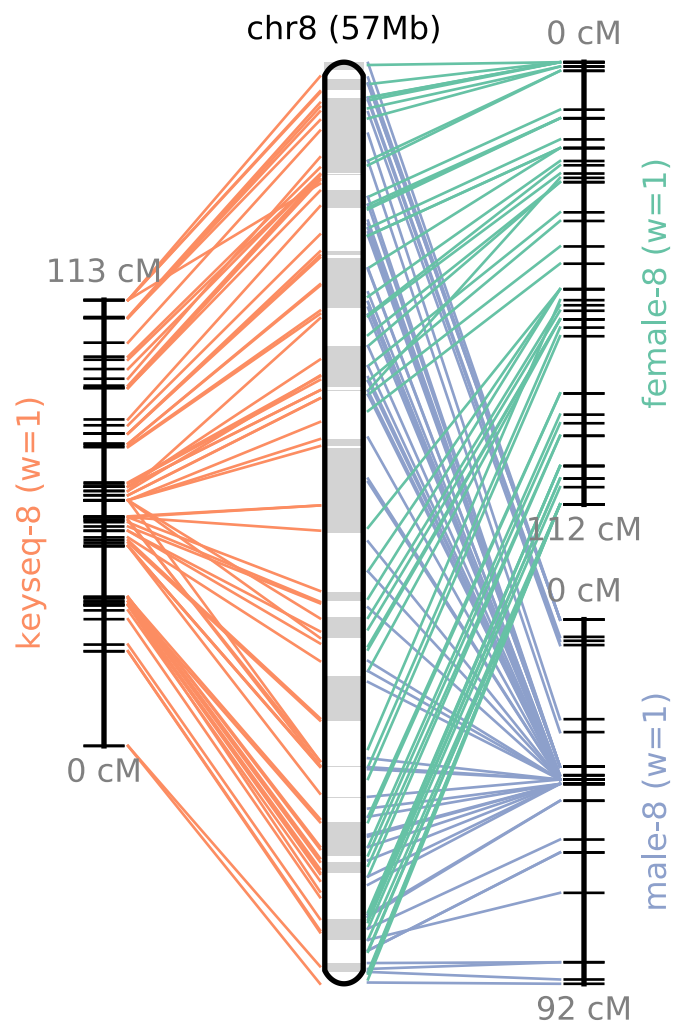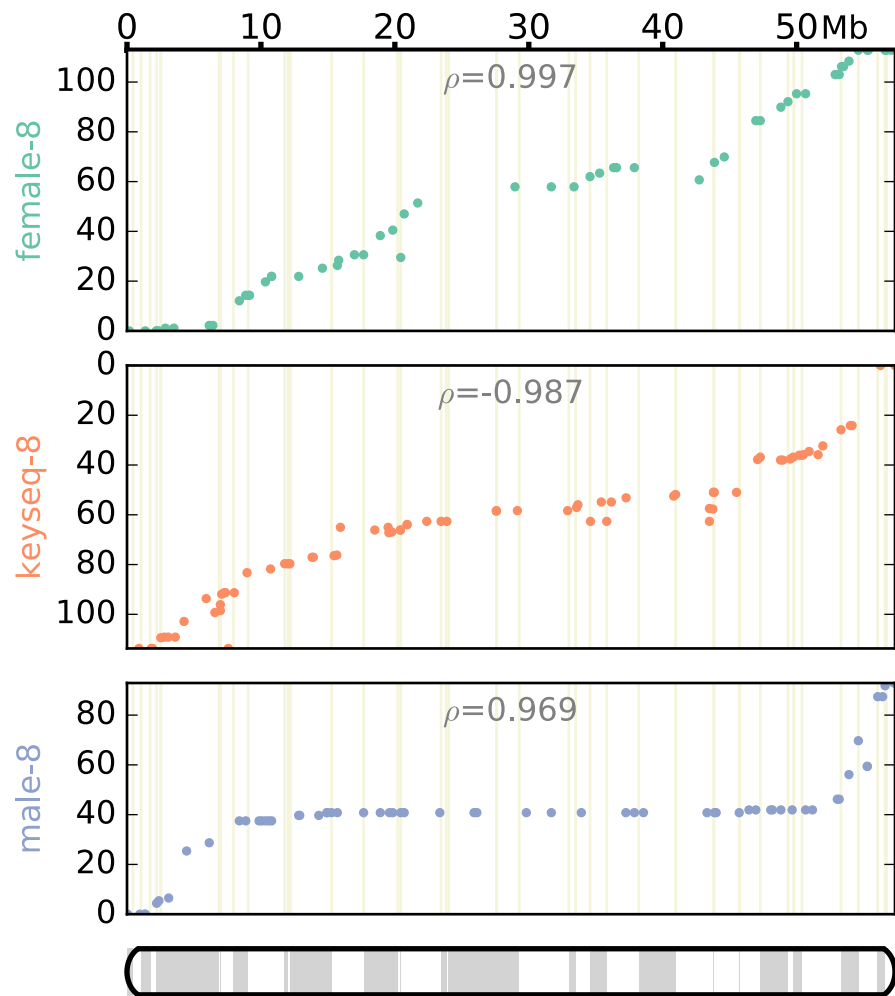

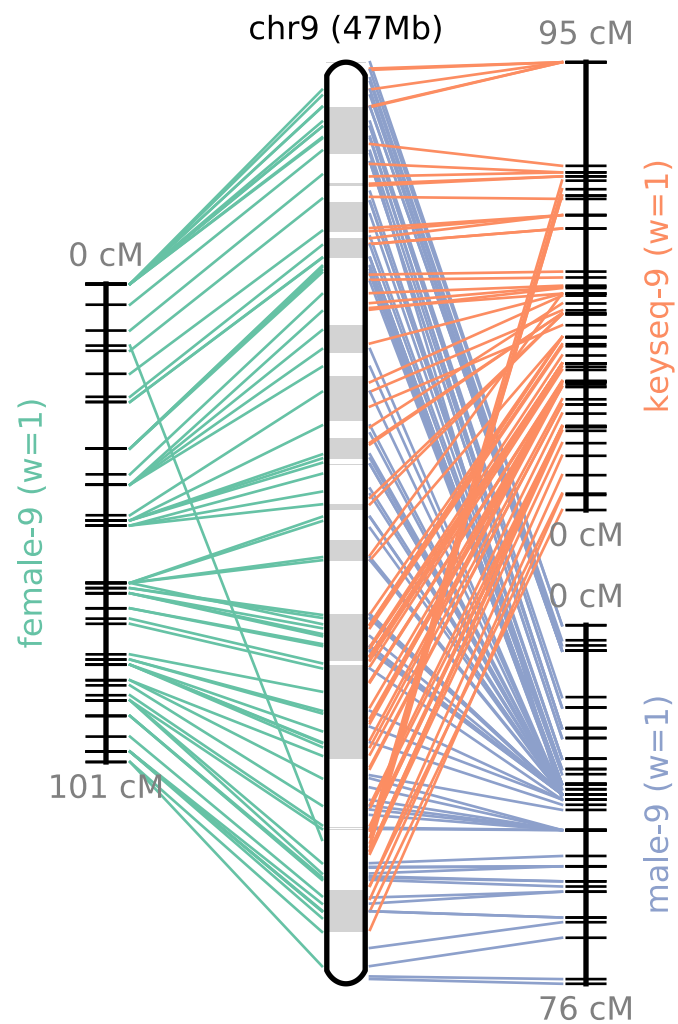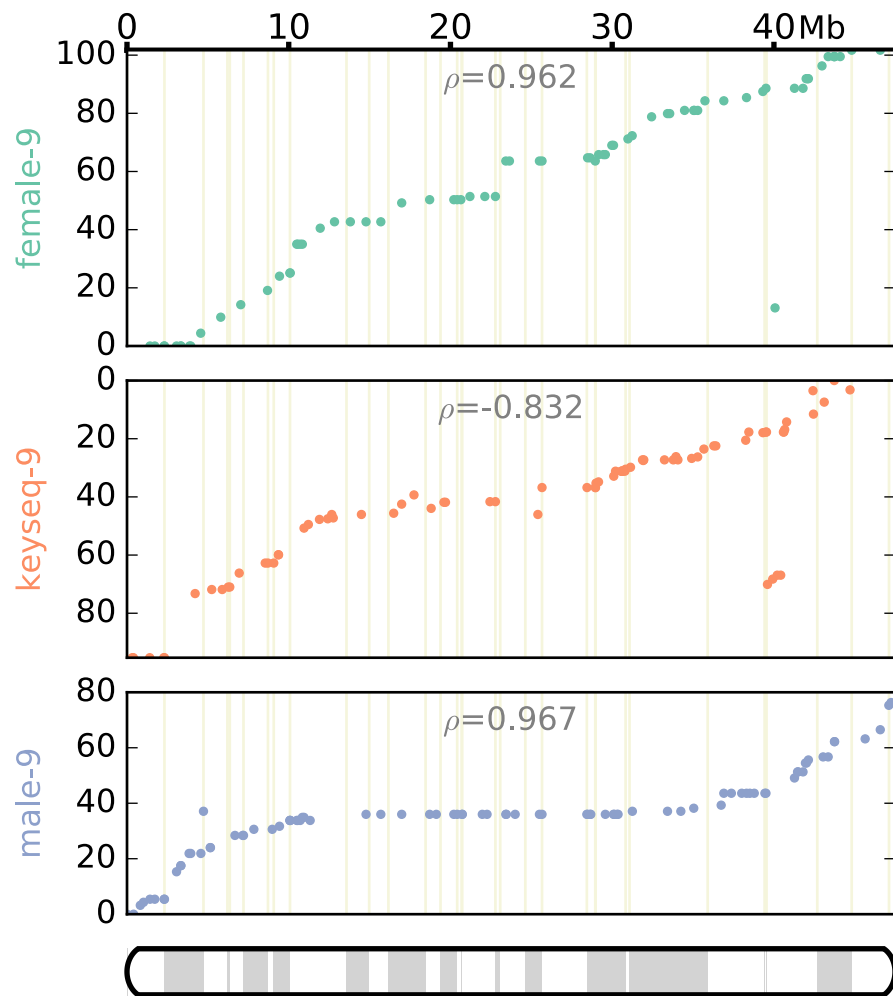

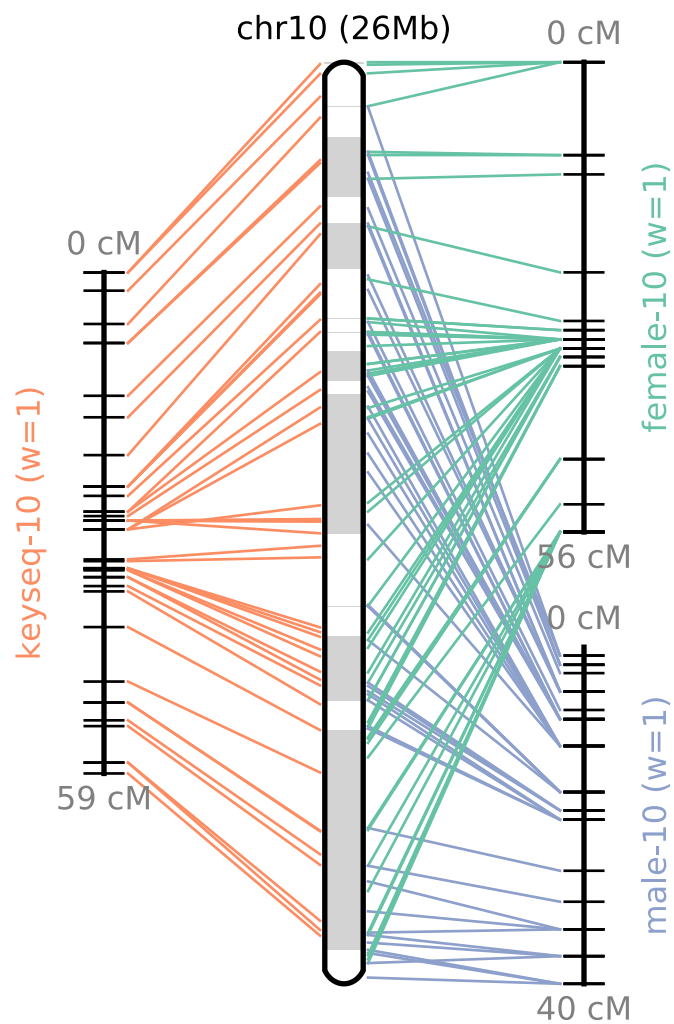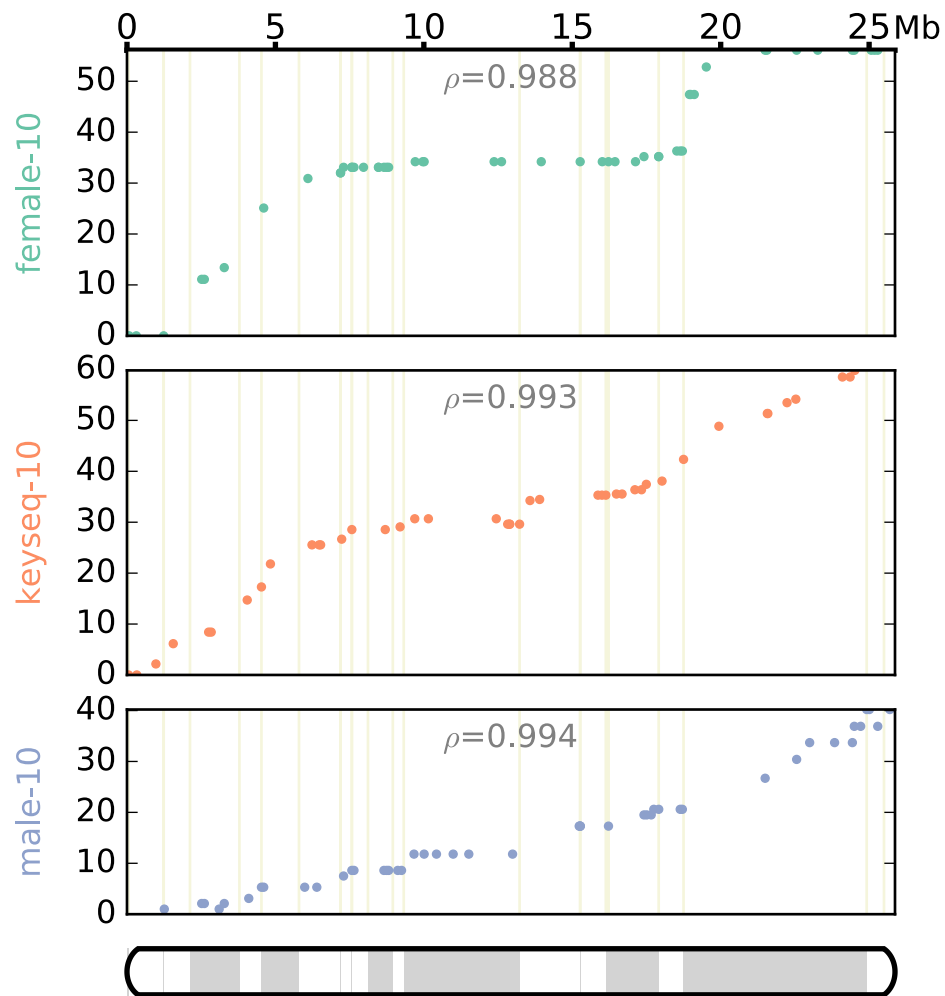

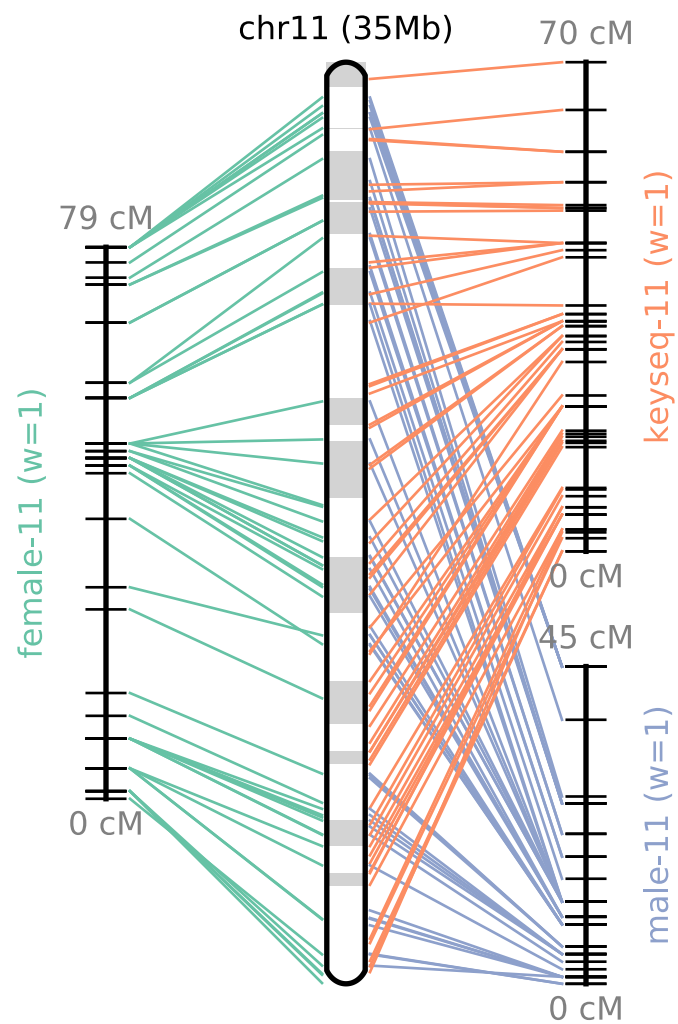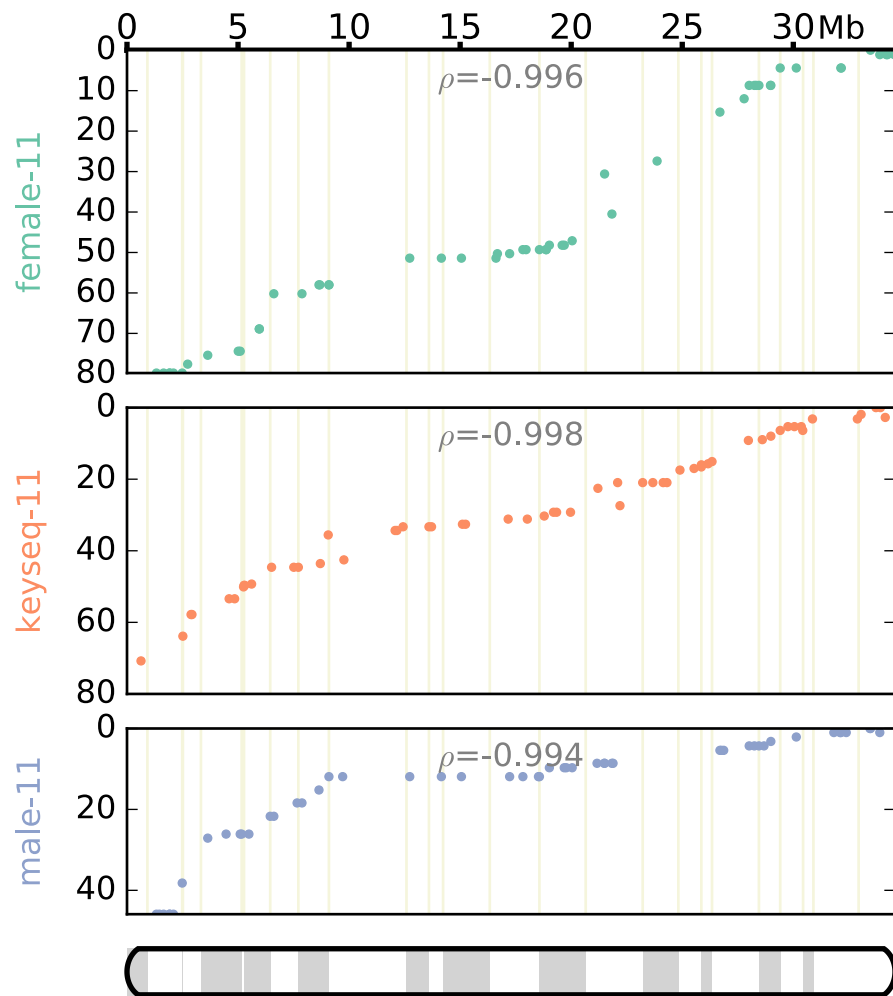

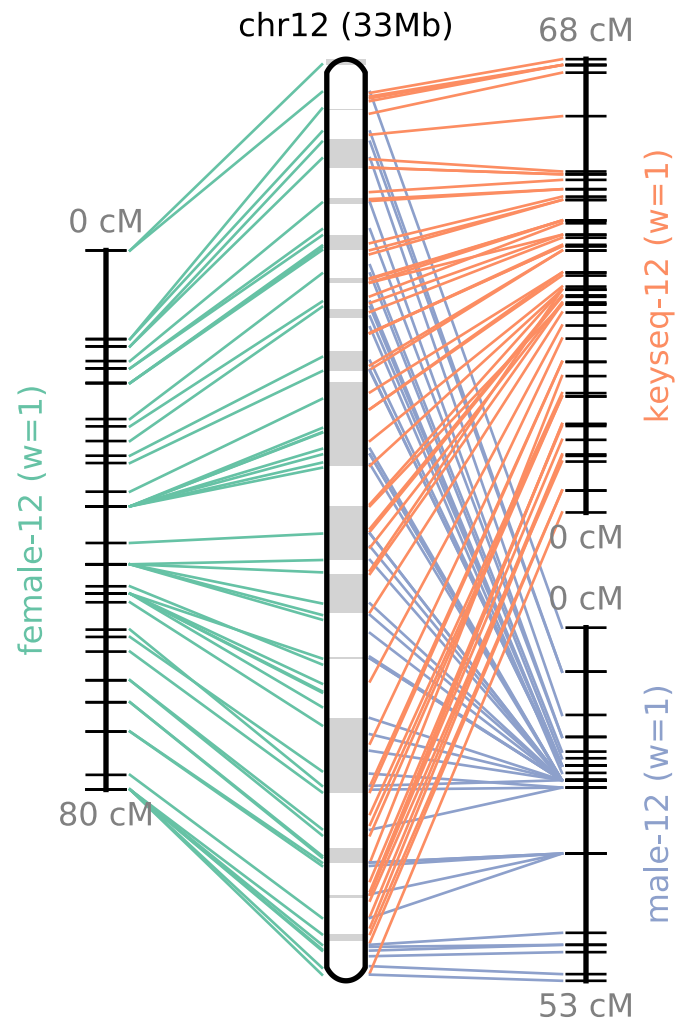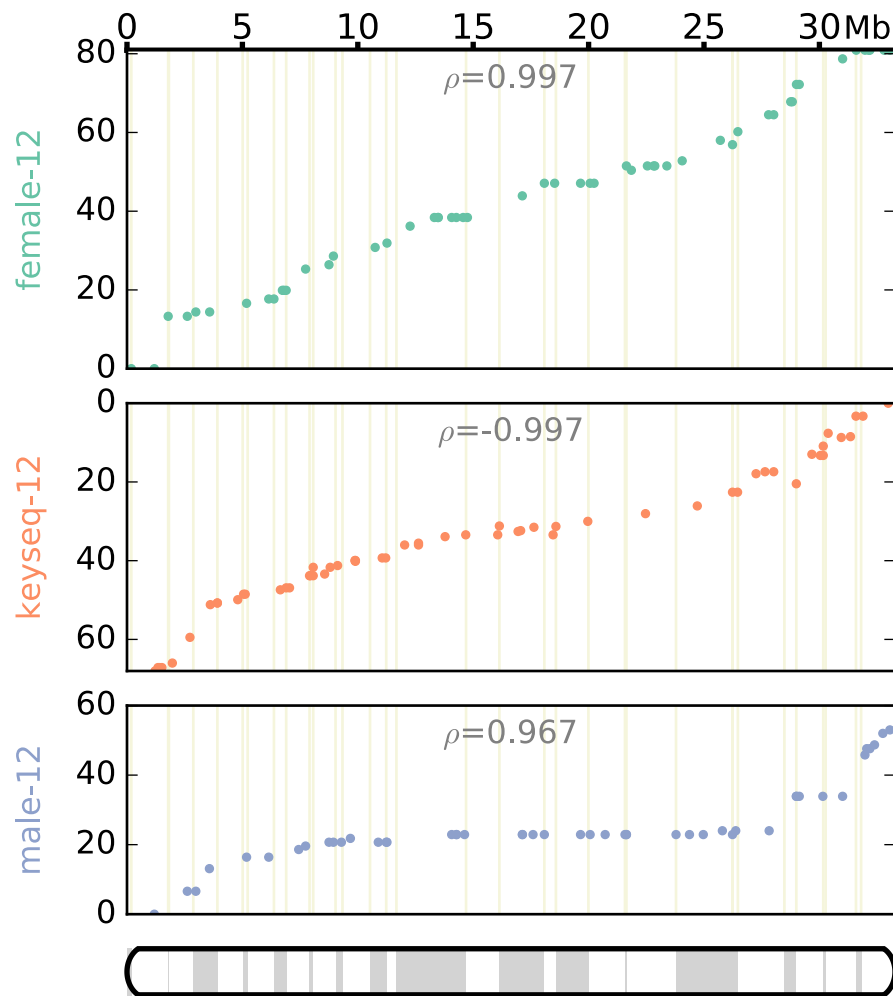

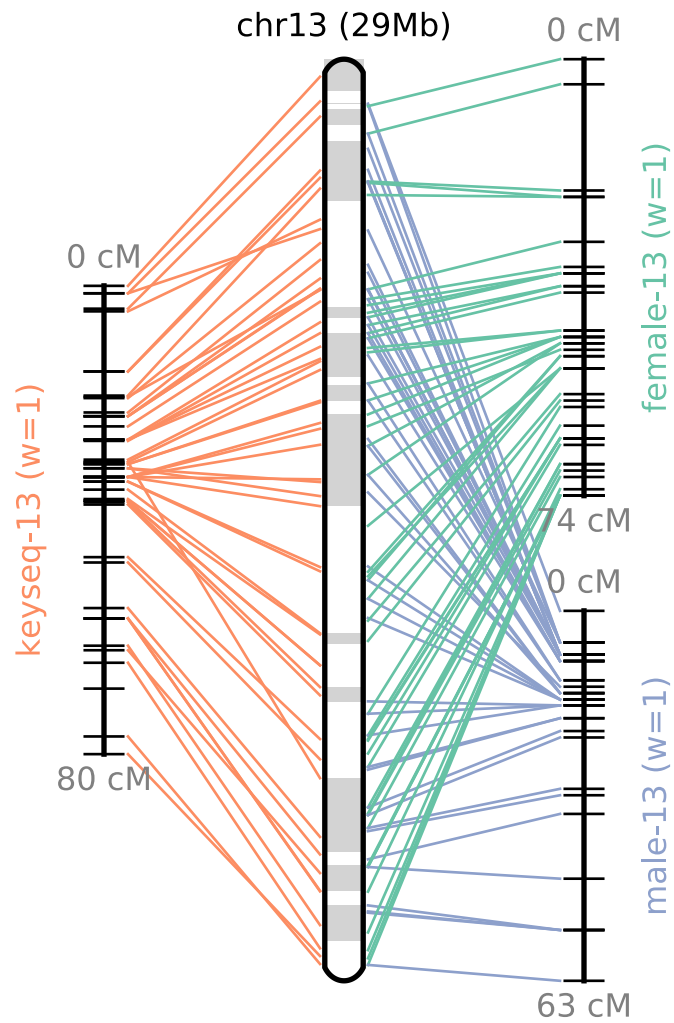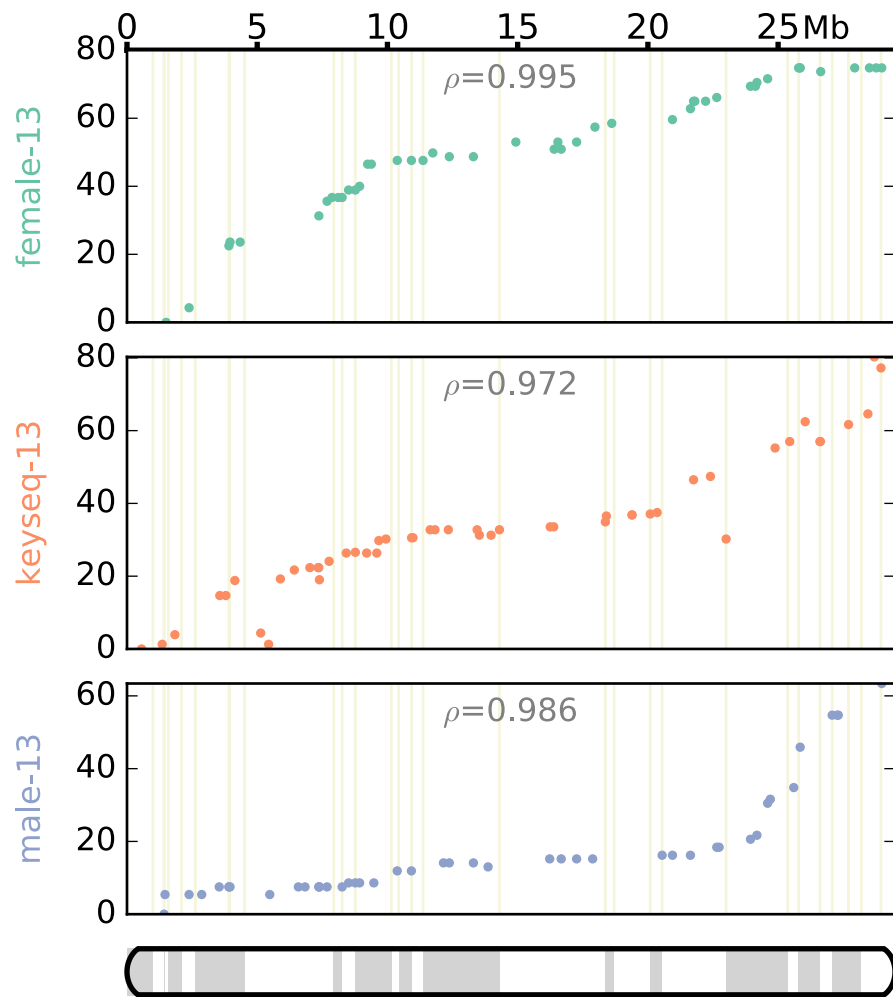

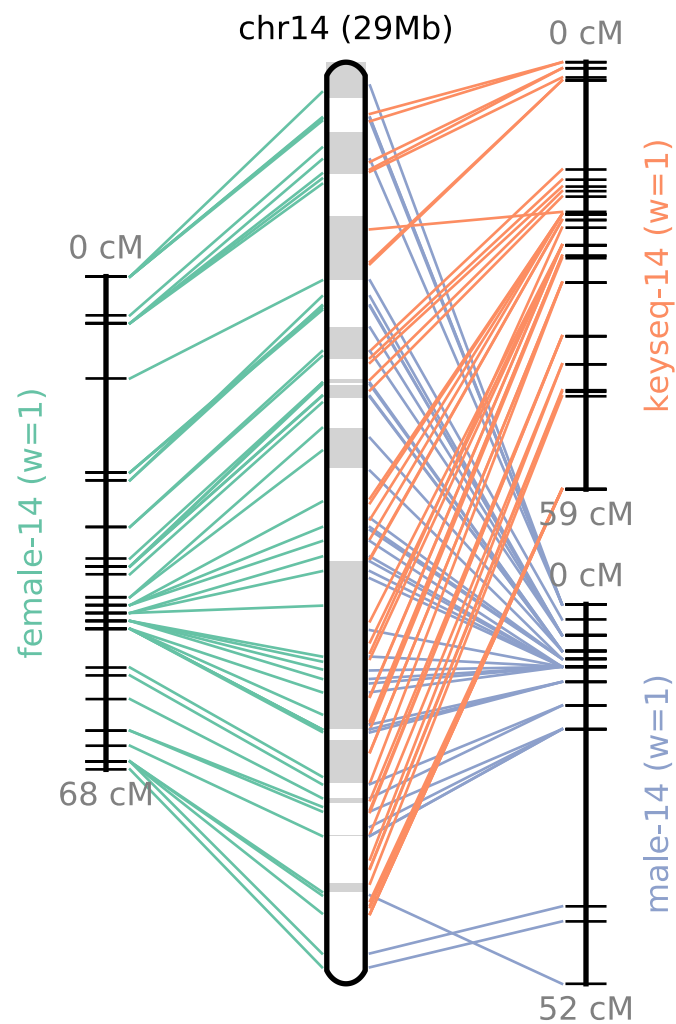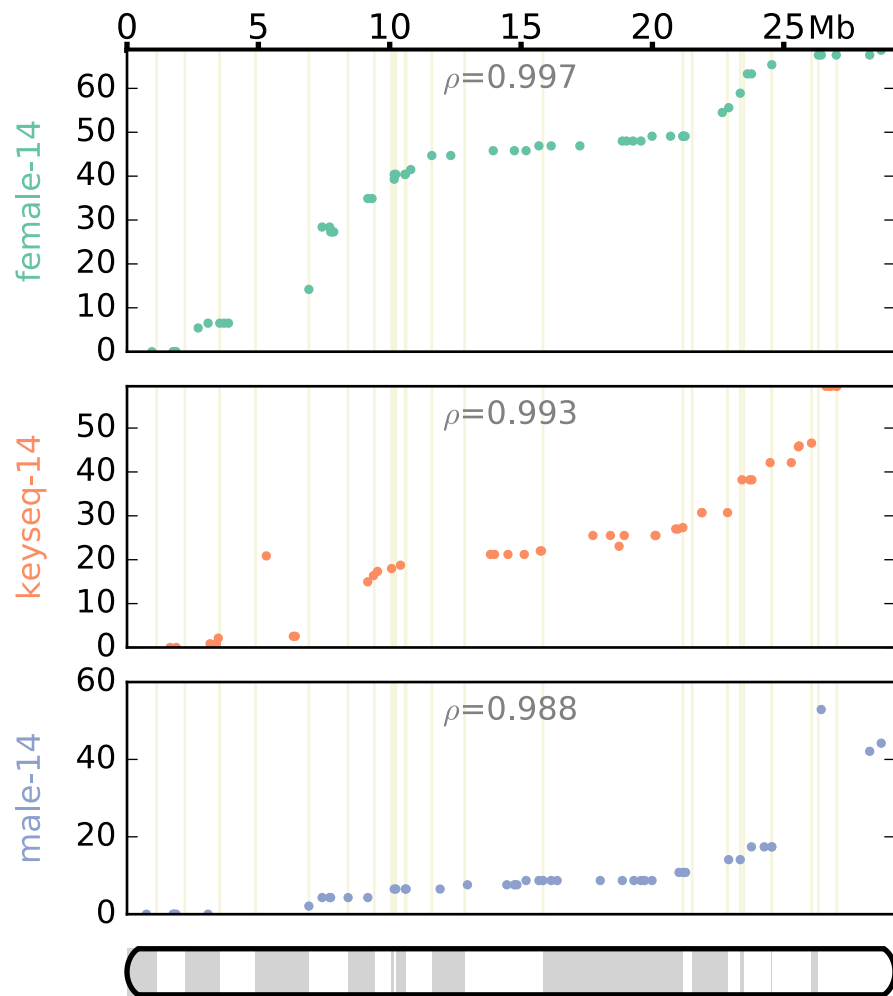

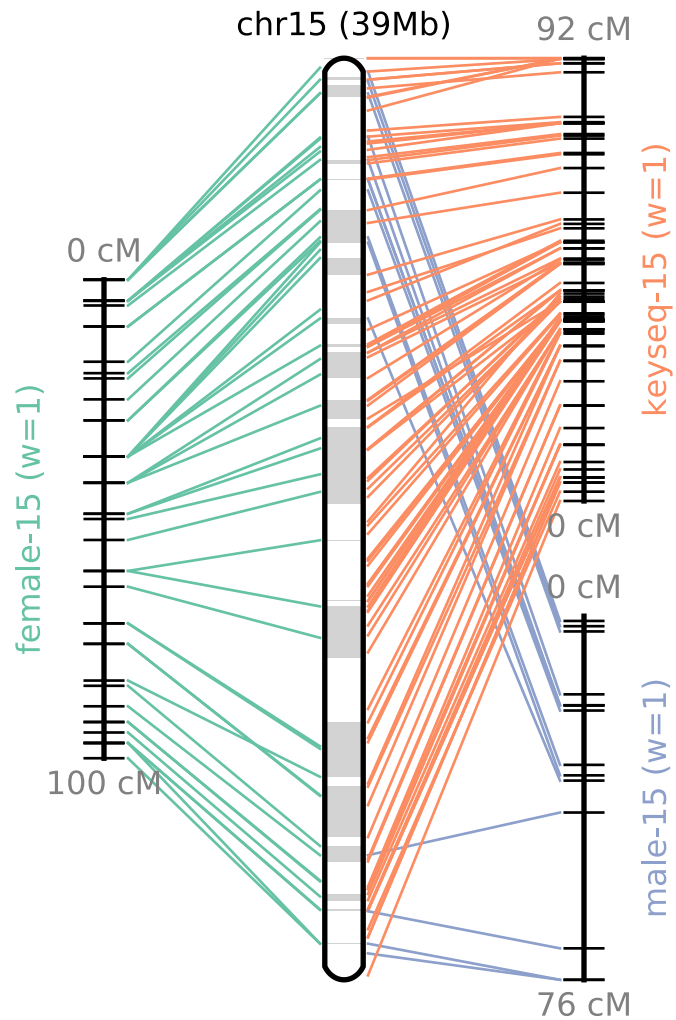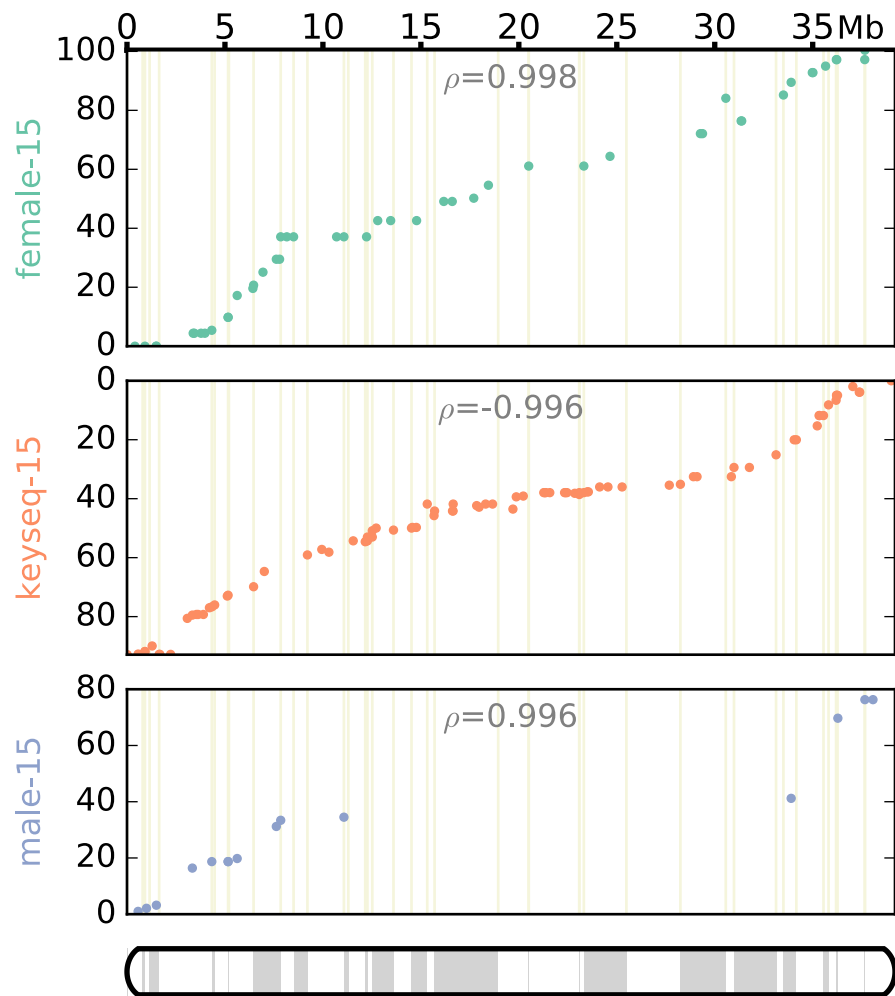

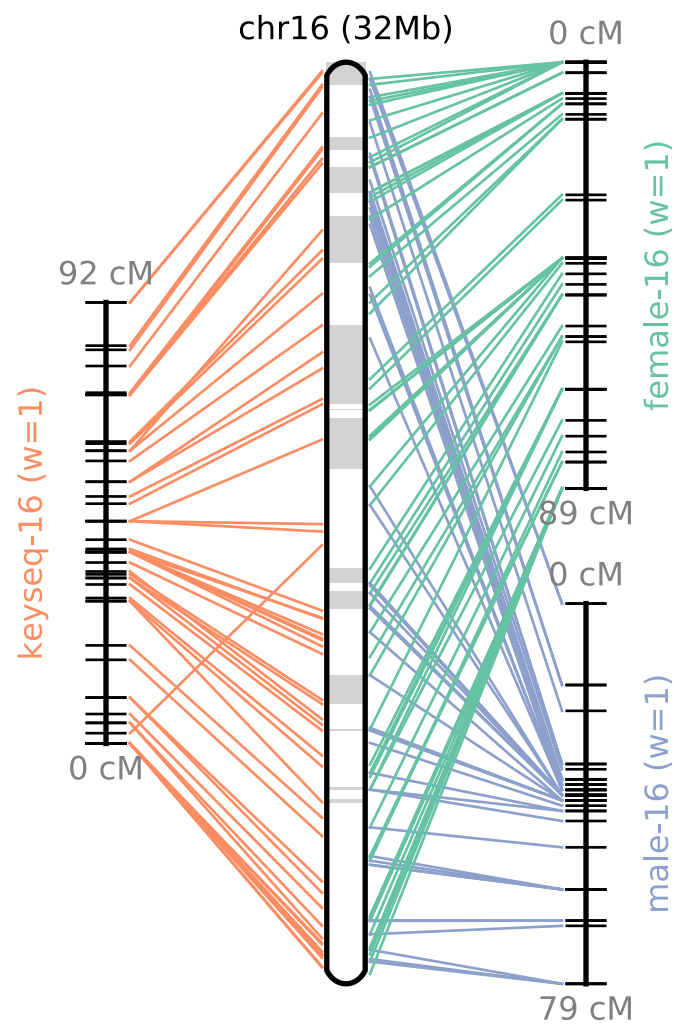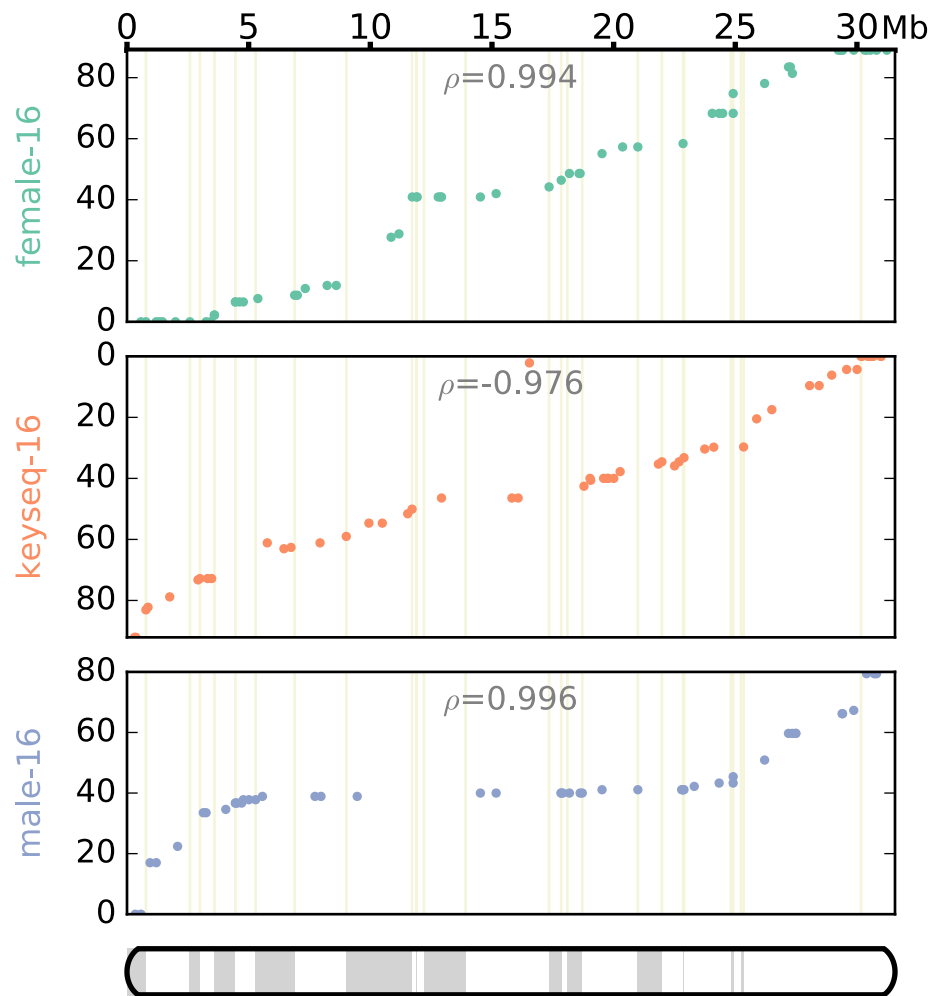

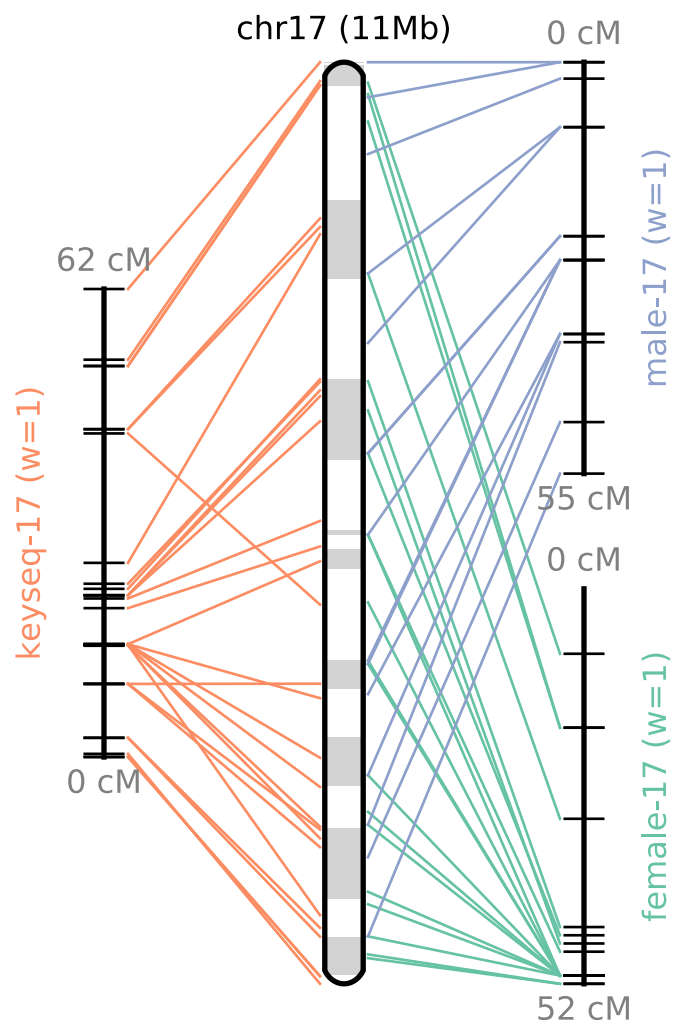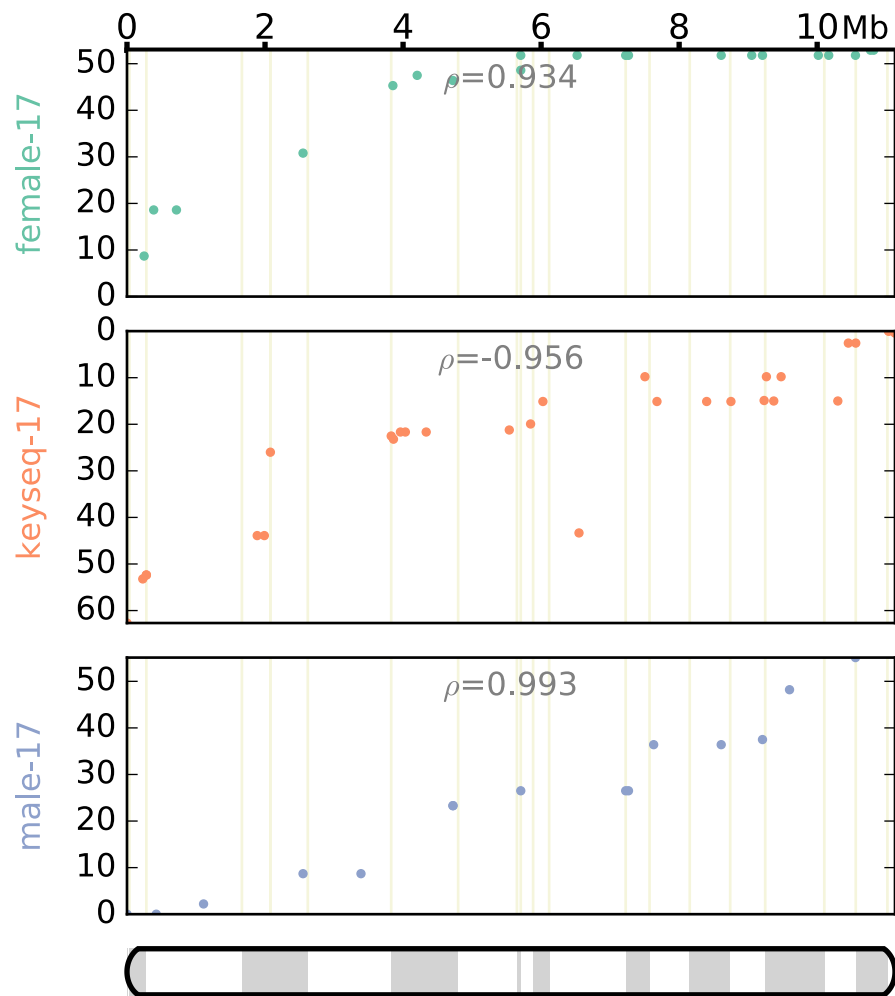

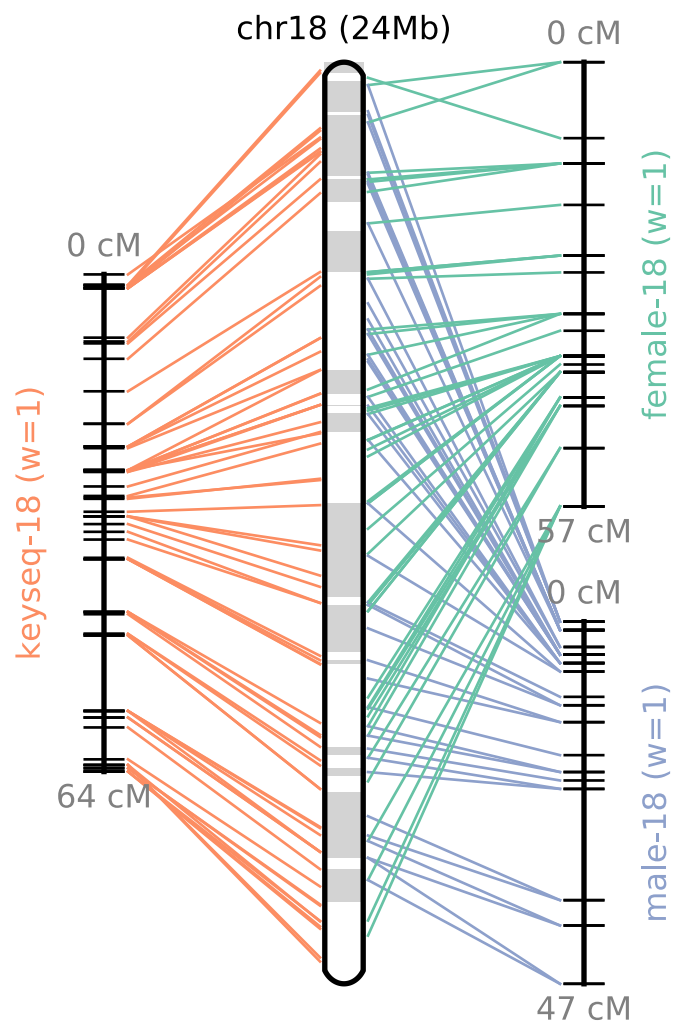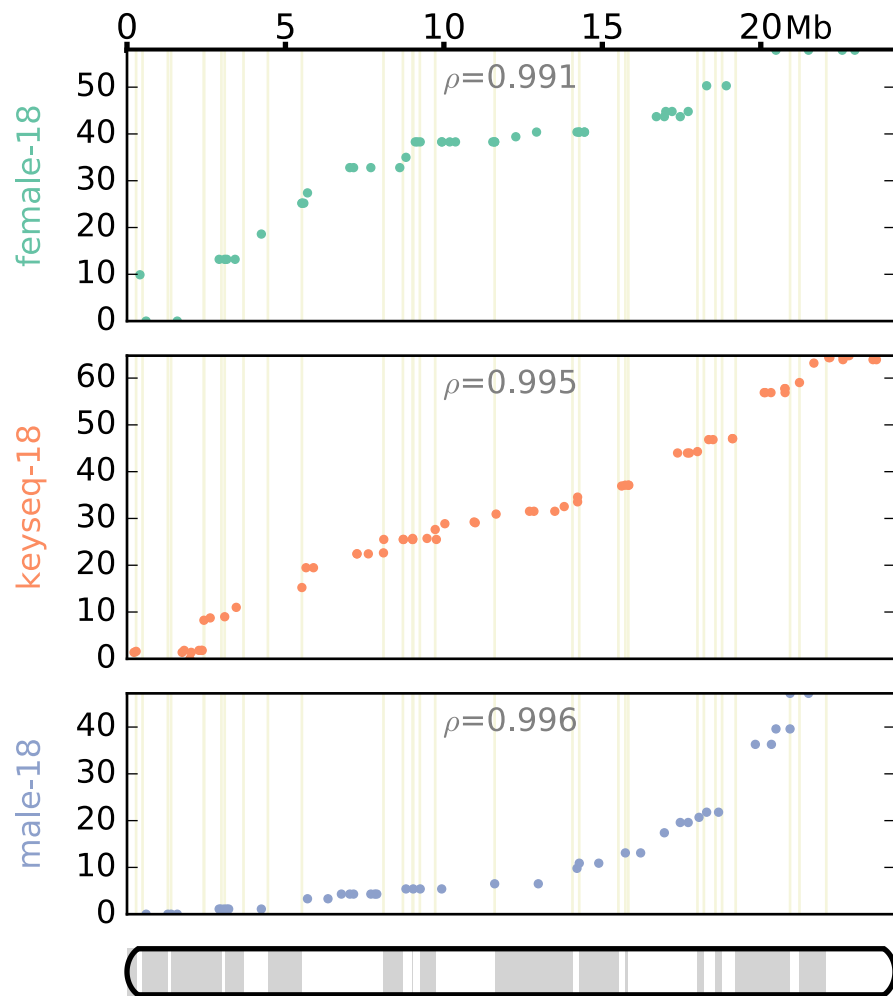

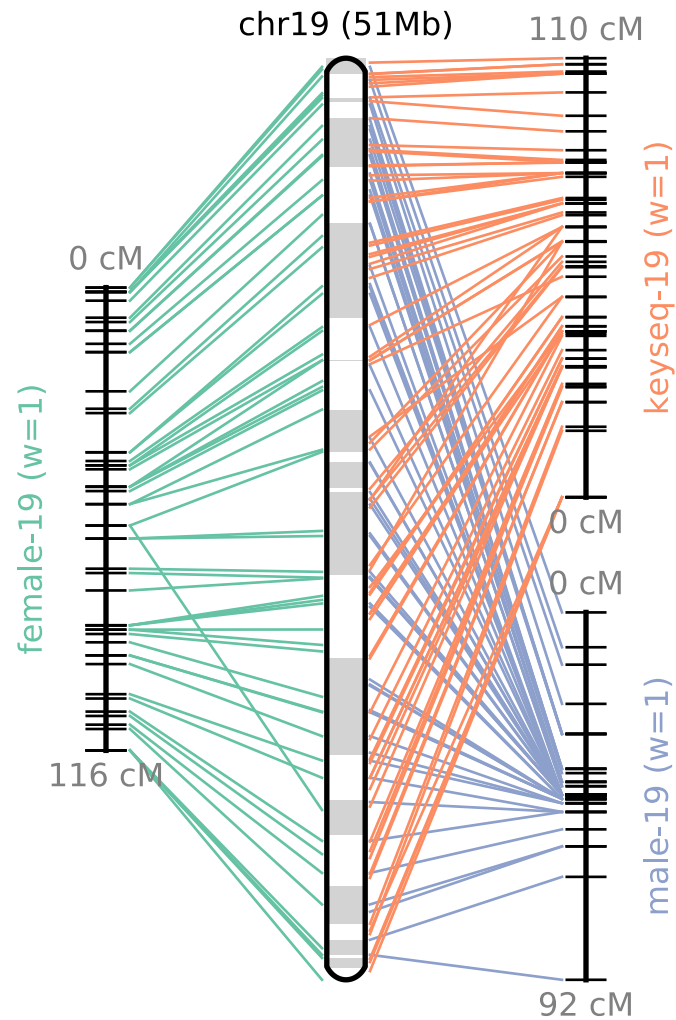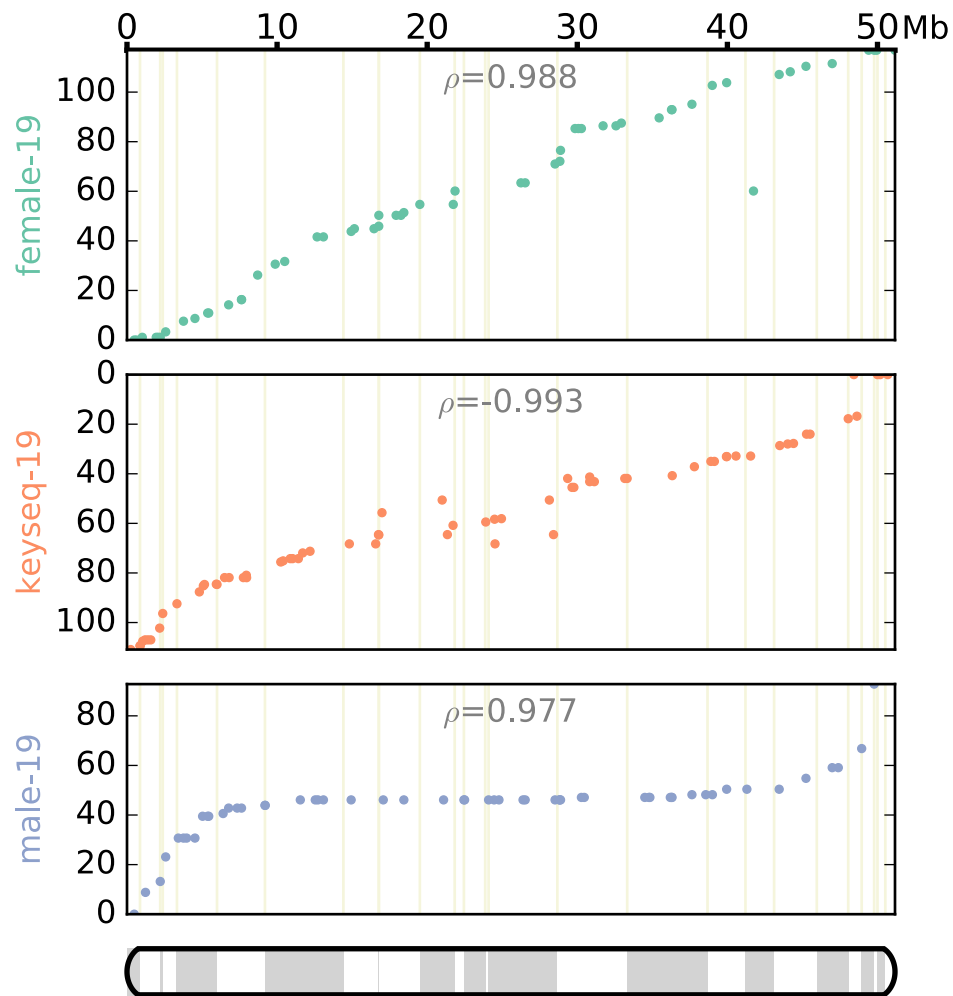

Supplement: S1 File — (PDF) [file pone.0201784.s009.pdf]
